# Rare Solid Pancreatic Lesions on Cross-Sectional Imaging

**DOI:** 10.3390/diagnostics13162719

**Published:** 2023-08-21

**Authors:** Ana Veron Sanchez, Nuria Santamaria Guinea, Silvia Cayon Somacarrera, Ilias Bennouna, Martina Pezzullo, Maria Antonietta Bali

**Affiliations:** 1 Hôpital Universitaire de Bruxelles, Institut Jules Bordet, 1070 Brussels, Belgium; ilias.bennouna@hubruxelles.be (I.B.);; 2 Clatterbridge Cancer Centre, Liverpool L7 8YA, UK; 3 Hospital Universitario Marques de Valdecilla, 39008 Santander, Spain; 4 Hôpital Universitaire de Bruxelles, Hôpital Erasme, 1070 Brussels, Belgium

**Keywords:** pancreas, solid, rare

## Abstract

Several solid lesions can be found within the pancreas mainly arising from the exocrine and endocrine pancreatic tissue. Among all pancreatic malignancies, the most common subtype is pancreatic ductal adenocarcinoma (PDAC), to a point that pancreatic cancer and PDAC are used interchangeably. But, in addition to PDAC, and to the other most common and well-known solid lesions, either related to benign conditions, such as pancreatitis, or not so benign, such as pancreatic neuroendocrine neoplasms (pNENs), there are solid pancreatic lesions considered rare due to their low incidence. These lesions may originate from a cell line with a differentiation other than exocrine/endocrine, such as from the nerve sheath as for pancreatic schwannoma or from mesenchymal cells as for solitary fibrous tumour. These rare solid pancreatic lesions may show a behaviour that ranges in a benign to highly aggressive malignant spectrum. This review includes cases of an intrapancreatic accessory spleen, pancreatic tuberculosis, solid serous cystadenoma, solid pseudopapillary tumour, pancreatic schwannoma, purely intraductal neuroendocrine tumour, pancreatic fibrous solitary tumour, acinar cell carcinoma, undifferentiated carcinoma with osteoclastic-like giant cells, adenosquamous carcinoma, colloid carcinoma of the pancreas, primary leiomyosarcoma of the pancreas, primary and secondary pancreatic lymphoma and metastases within the pancreas. Therefore, it is important to determine the correct diagnosis to ensure optimal patient management. Because of their rarity, their existence is less well known and, when depicted, in most cases incidentally, the correct diagnosis remains challenging. However, there are some typical imaging features present on cross-sectional imaging modalities that, taken into account with the clinical and biological context, contribute substantially to achieve the correct diagnosis.

## 1. Introduction

In addition to the most common solid pancreatic lesions related to benign conditions, such as chronic pancreatitis, or to malignancy mainly represented by PDAC and pNENs, there are several rare pancreatic solid lesions that can be very challenging to correctly diagnose due to knowledge scarcity secondary to their very low incidence.

A variety of epithelial tumours may arise within the pancreas, with ductal, acinar and neuroendocrine differentiation. In addition, most of the mesenchymal tumours found in extrapancreatic locations may also arise within the pancreas. However, in cases such as the solid pseudopapillary neoplasm, there is no defined cell lineage identified.

These lesions can present a benign, potentially malignant and malignant behaviour and may show typical and atypical imaging features on cross-sectional imaging modalities. Combining these imaging findings with epidemiological, clinical and biological data may contribute to achieving the correct diagnosis.

Table 1 reports the rare solid pancreatic lesions classified in three sections based on their behaviour: benign, potentially malignant and malignant.

Due to their rarity, statistical data regarding the incidence and prevalence are not easy to find and published literature about these pancreatic lesions mainly consists of case reports or series. Table 2 reports incidence/prevalence data of these rare solid pancreatic lesions. Therefore, the aim of this pictorial review is to gather rare solid lesions that can be encountered in the pancreas and describe the cross-sectional imaging features, highlighting their respective hallmarks, with a focus on differential diagnosis and on patient management.

Concerning image acquisition, it is crucial to note the importance of including a pancreatic parenchymal phase, obtained 35–40 s after intravenous contrast administration, as it ensures a relatively increased enhancement of the pancreatic parenchyma and shows higher differences in attenuation between normal parenchyma and hypovascular tumours, as well as allowing assessment of arteries [1]. This parenchymal phase is followed by a portal venous phase, obtained at 70 s, to assess the veins, as venous flow artifacts observed in the pancreatic phase will be avoided [1]. In addition, hepatic enhancement will be increased and metastases will be detected more easily. Dynamic study finishes with a delayed venous phase, at 180 s.

## 2. Benign Lesions (Table S1)

### 2.1. Intrapancreatic Splenic Tissue (Figure 1)

Intrapancreatic splenic tissue (IPST) may occur under the form of accessory spleen or splenosis. Accessory spleens are congenital abnormalities, in which the earliest forms of spleen fail to fuse during the fifth week of embryonic life [2] and are usually located next to their embryonic origin or along their migration path [3]. Splenosis, though, is an acquired condition, in which a heterotopic transplantation of splenic tissue takes place [4], frequently after spleen surgery or trauma. It can be found anywhere throughout the abdomen, the pelvis and even the chest [5], although it occurs most frequently in the liver [6] and is rare within the pancreas [7]. Sixty-one accessory spleens were found within the pancreatic tail in a 3000-patient autopsy study [8].

The pancreatic tail is a preferred IPST location, either in the form of IPAS or splenosis [9] and it has been described as the second most common site of accessory spleen [10].

IPST commonly appears incidentally on cross-sectional techniques as a well-defined nodule, presenting clear demarcated borders with the adjacent parenchyma. It shows the same signal intensity as the spleen, with the same behaviour following intravenous contrast administration, heterogeneously enhancing in a zebra-pattern, during the arterial phase [11] due to different flow rate of contrast through the red and white pulp [12], and becoming homogeneous during the portal phase. However, this heterogeneous enhancement may be missing, especially in small lesions [11]. An elevated signal intensity in diffusion-weighted images (DWIs) using a high b-value is also suggestive of IPST [13]. IPST may grow and potentially mimic malignancy [14].

Spleen surgery or trauma history may be very helpful to achieve a correct diagnosis that is crucial to avoid unnecessary surgery or biopsy.

IPST should be included in the differential diagnosis of pancreatic hypervascular lesions, namely pNENs, solid pseudopapillary tumour (SPT) and pancreatic metastasis (PM) from renal clear cell carcinoma (RCC). Epidermoid cyst and inflammatory pseudotumour have been described as associated with IST [15,16,17], and the diagnosis under these circumstances may be challenging.

Tc-99m-labelled heat-denatured red blood cells (Tc-99m-DRBCs) are currently the gold standard technique to specifically prove the diagnosis of IST [18], as Tc-99m-DRBCs are trapped by reticuloendothelial cells.

**Figure 1:**
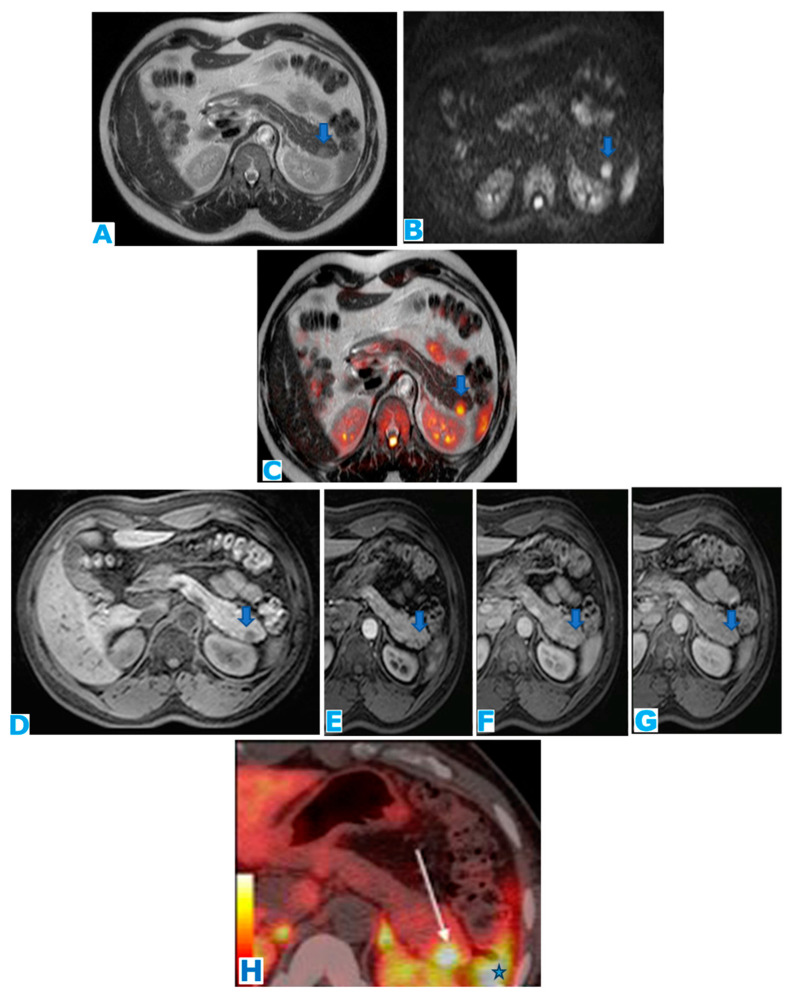
(**A**): axial T2-weighted images (T2WIs), (**B**): DWI, (**C**): axial T2WI-DWI fused images, (**D**): non-contrast-enhanced (NCE) fat-saturated (FS) T1-weighted images (T1WI), (**E**–**G**): axial contrast-enhanced (CE) dynamic FST1WI, (**E**): pancreatic parenchymal phase, (**F**): portal venous phase, (**G**): delayed venous phase, (**H**): Ga-68-DOTATOC PET-CT. Incidentally discovered IPAS in a 56 year-old patient, during check-up examination for elevation of pancreatic enzymes. Note a slightly hyperintense lesion within the pancreatic tail in T2WI (arrow in (**A**)), with diffusion restriction (arrow in (**B**,**C**)), conspicuous in the unenhanced phase (arrow in (**D**)) but not so much following intravenous contrast administration (arrow in (**E**–**G**)). Endoscopic ultrasound-guided fine-needle aspiration (EUS-FNA) obtained small epithelioid cells, with antichromogranin- and synaptophysin-positive immunostaining and concluded a pNEN grade II (Ki 67 = 5%). However, in the PET-CT, the lesion (arrow in (**H**)) showed the same uptake as the spleen (* in (**H**)) and no non-physiological uptake was found, so an IPAS was suspected on imaging. The FNA result was a false positive for NEN secondary to contamination of normal neuroendocrine pancreatic tissue as the patient underwent a left-sided pancreatectomy with spleen preservation and the histological examination concluded IPAS.

### 2.2. Pancreatic Tuberculosis (Figure 2)

Pancreatic tuberculosis (PT) occurs very rarely, predominantly during a multiorgan abdominal spread of the infection [19]. Only 116 cases have been reported in the literature [20]. When isolated, its diagnosis is not suspected and is frequently achieved after histologic examination, following resection [21]. It has been theorised that pancreatic enzymes serve as shields against *Mycobacterium tuberculosis* [22].

In the western world, PT occurs mainly in immunocompromised patients [21,23]. It seems to be more frequent between the fourth and fifth decades of life [24,25]. Gender association is not clear [26,27].

The most frequent clinical presentations are vague non-specific symptoms (fatigue, fever, weight loss, nausea and vomiting) [28] or a history of acute or chronic pancreatitis [21]. Less frequently, it can also present as obstructive jaundice or gastrointestinal bleeding [29].

The body of the pancreas seems to be the favoured location, closely followed by the head [25,30].

Presentation patterns are focal masses [31], multiple small nodules [23] and, less frequently, a diffuse involvement, mimicking an acute pancreatitis [32], with increased signal intensity in T2-weighted images (T2WIs) [21].

Focal pattern PT may appear as a well-defined cystic–solid mass, with varying aspects depending on the proportion of cystic and solid components [23]: hypodense on CT, hypo- or isointense in T1-weighted images (T1WIs) and heterogeneous in T2WIs. After intravenous contrast administration, peripheral enhancement with central necrosis or enhancing solid components may be depicted [25]. When predominantly cystic, PT may be misdiagnosed as a cystic tumour, such as a cystadenoma, a pseudocyst in the setting of chronic pancreatitis or an infected abscess. If, on the other hand, PT consists of a mainly solid lesion associated with biliary or main pancreatic duct (MPD) dilatation, it may be indistinguishable from PDAC (especially if accompanied by peripancreatic lymph nodes and signs of vascular invasion), lymphoma and metastasis [33].

Calcifications are frequently encountered [34]. Dilatation of the bile and pancreatic ducts may occur, though infrequently, despite the mass effect on the ducts [28]. Displacement and stenosis of an otherwise normal MPD are frequent features, without much prestenotic dilatation [34]. Vascular invasion has been described [25,35].

As lymph nodes are the most common tuberculosis site within the abdomen, accompanying peripancreatic lymphadenopathy is frequently found [36], mostly showing peripheral enhancement with central low attenuation, corresponding to granulation tissue encircling central caseous necrosis [37]. This appearance, although highly suggestive, is not pathognomonic of PT.

Both cytology and histological examination following imaging-guided fine-needle aspiration (FNA) or biopsy (FNB), respectively, are the gold standard diagnosing techniques. PT can be effectively treated with antituberculous therapy [38].

**Figure 2:**
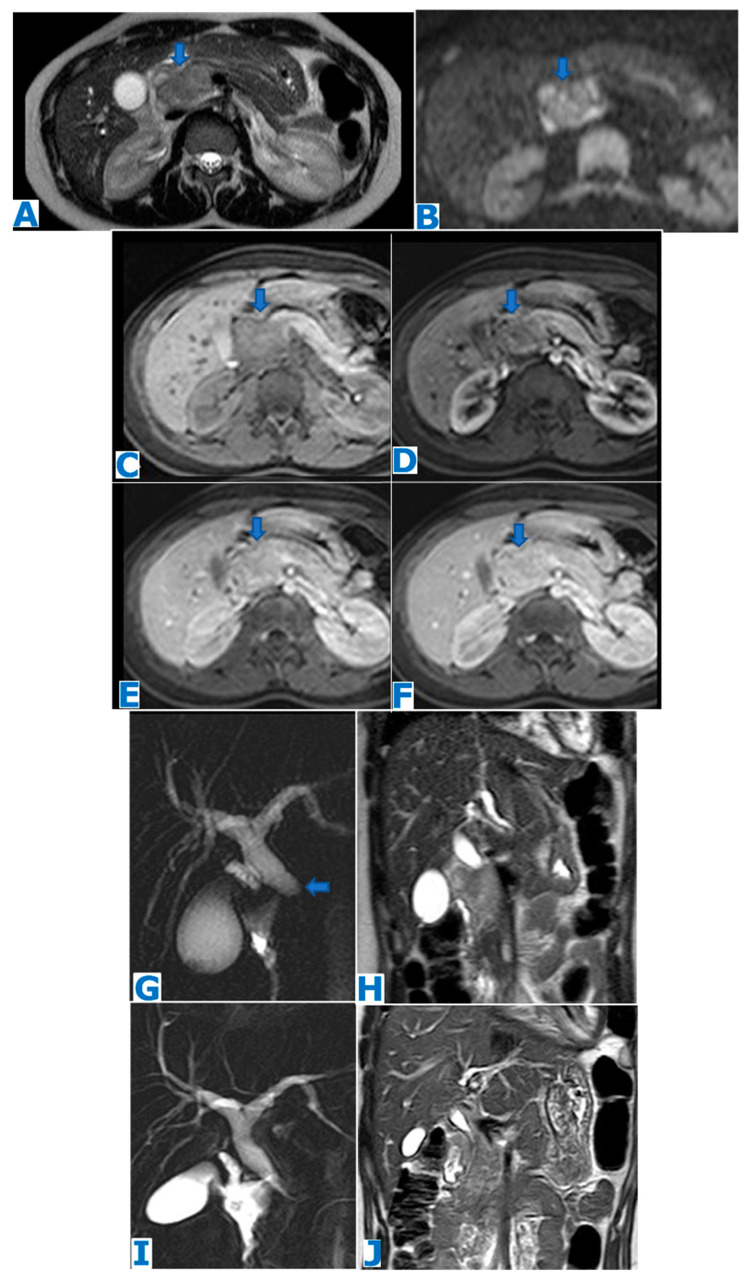
(**A**): axial T2WI, (**B**): DWI, (**C**): NCE FST1WI, (**D**–**F**): axial CE dynamic FST1WI (**D**): pancreatic parenchymal phase, (**E**): portal venous phase, (**F**): delayed venous phase, (**G**): MCRP, (**H**): coronal T2WI, (**I**): posttreatment MCRP, (**J**): posttreatment coronal T2WI. Primary pancreatic tuberculosis in a 15-year-old patient from Burundi presenting with abdominal pain and anicteric cholestasis. Note in the T2WI a hyperintense mass in the head of the pancreas (arrow in (**A**)) causing an abrupt biliary duct cutoff (arrow in (**G**)) and upstream dilatation. The mass shows diffusion restriction (arrow in (**B**)) and progressive enhancement in the dynamic sequences (arrows in (**C**–**F**)). EUS-guided FNA revealed necrosis, Langhans giant cells, lymphocytes and macrophages organised in granulomas. Thoracic radiography (not shown) was normal, and diagnosis was primary pancreatic tuberculosis. Both the lesion and mass effect on the common bile duct completely resolved after treatment (**I**,**J**).

### 2.3. Solid Serous Cystadenoma (Figure 3)

Solid serous cystadenoma (SSCA) is the rarest variant of pancreatic serous cystadenoma, accounting for only 3% of all cases [39], and with only 22 cases reported in the literature [40]. Serous cystadenomas are benign tumours, usually composed of cysts that can measure up to 2 cm, with a typical honeycomb appearance. A central scar, often calcified, is frequently identified [41].

The solid variant is frequently misdiagnosed, because cystic spaces are either absent or scarce and too tiny [42]. In addition, serous cystadenomas may contain intratumoral haemorrhage, which adds to the high density of these lesions, contributing to the solid appearance. It occurs most commonly in elderly women, as an incidental finding, with no site of preference [40]. If symptomatic, the presentation is usually non-specific, with abdominal pain, abdominal mass and, rarely, jaundice [43].

As the remaining serous cystadenomas, SSCAs are well-delimited lesions, hypointense in T1WIs and hyperintense in T2WIs [44]. Its most salient feature is an early rapid enhancement followed by isointensity in the portal phase, a fact that frequently leads to a misdiagnosis of pNEN [40,45,46]. T2WIs and especially MR cholangiopancreatography (MRCP), a heavily T2WI sequence with an echo time 10 times longer than that of regular T2WIs, help diagnose the hyperintense cyst [47]. A mild dilatation of the pancreatic duct may happen, due to compression.

Preoperative diagnosis is challenging, and aside from pNEN, it is also commonly mistaken for SPT, PM and even PDAC [47].

Once the diagnosis is suspected at cross-sectional imaging, a confirmation by EUS-FNA is achieved in only half of patients [42], as SSCA’s nature may cause the sample to lack the epithelial tissue required for diagnosis.

As in typical serous cystadenomas, surgery is only recommended when causing symptoms, due to compression of neighbouring organs [48] or if diagnosis remains uncertain after workup [43].

**Figure 3:**
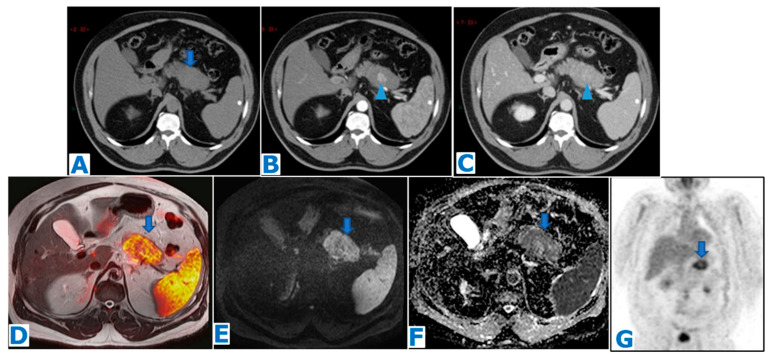
(**A**): axial NCE-CT, (**B**): axial CE pancreatic parenchymal phase CT, (**C**): axial CE portal venous phase CT, (**D**): axial T2WI-DWI fusion, (**E**): DWI, (**F**): ADC, (**G**): coronal FDG-PET. Solid serous cystadenoma. A 67-year-old patient with fatigue and abdominal pain, referred from another institution with the diagnosis of pancreatic neoplasm. CA 19.9 within normal limits. CT shows a solid lesion in the body of the pancreas (arrow in (**A**)), with central enhancement in the arterial phase (arrowhead in (**B**)), which persists during portal phase (arrowhead in (**C**)). There is no downstream MPD dilatation. Note the diffusion restriction (arrow in (**D**–**F**)) and the peripheral hypermetabolic uptake on the PET-CT (arrow in (**G**)). EUS-guided FNB only obtained inflammatory cells, with no evidence of malignancy. The lesion remained suspicious, and the patient underwent a left pancreatectomy. Histological examination revealed a SSCA.

## 3. Potentially Malignant Lesions (Table S2)

### 3.1. Solid Pseudopapillary Tumour (Figure 4 and Figure 5)

SPT is a rare pancreatic neoplasm accounting for 2% of all exocrine pancreatic neoplasms [49]. It is an epithelial tumour, but its pathogenesis remains unclear as its cells of origin are unlike any other cell found within the embryonic or adult pancreas [50]. It has been hypothesised that it arises from pluripotential embryonic stem cells [51].

SPT occurs tenfold more frequently in women than in men and this has given origin to a hypothesis linking the tumour to female sex hormones [51,52] or pointing to genital ridges close to the pancreatic anlage during organogenesis as a possible origin [53]. Published cases occurring in men report usually an older age and curiously an aggressive behaviour [54]. Its target populations are women younger than 40 years old [55].

There is no association with a functional endocrine syndrome [56] or with any laboratory finding [57].

This tumour grows at a slow rate, thus it does not cause symptoms and it is incidentally discovered in about 15% of patients [55,56]. When present, symptoms are non-specific [50,55,58]. Jaundice happens very rarely [55]. Hemoperitoneum secondary to tumour rupture, either spontaneous or traumatic [59,60], has been described as a rare presentation.

As a result of its slow growth rate and soft nature, SPT usually presents with a large size at diagnosis (mean size 5 cm) [61].

The tail of the pancreas is a favoured location [50]. An extrapancreatic site of origin is possible, though rare [62,63,64,65,66].

MPD or biliary dilatation almost never occurs [67].

Distant metastases, usually present at the time of diagnosis, occur in about 15% of patients [68] and are predominantly hepatic, peritoneal or lymphatic [68,69,70].

SPT is depicted in cross-sectional images as a homogeneous solid lesion that, as it becomes larger, outgrows the blood supply and suffers degenerative changes. Formation of pseudopapillae occurs as loss of tissue takes place. The stalks of these pseudopapillae contain fragile blood vessels and, as a result, intralesional haemorrhage happens frequently [71,72,73]. All these events contribute to a heterogeneous appearance with variable solid and cystic components and intralesional haemorrhagic and necrotic parts [50]. Intralesional haemorrhagic traces are considered to be pathognomonic findings [74,75]. Internal fluid–fluid levels may also be identified [76]. The different components will be better depicted on MR thanks to its high contrast resolution. A pseudocapsule, reflecting the tumour slow growth, is almost always depictable in tumours larger than 3 cm, granting well-delineated borders. True to its fibrous nature, it is typically hypointense in both T1- and T2WIs and enhances moderately after intravenous contrast injection [76]. Dystrophic calcifications are found in up to 30% of cases [59], with a variety of patterns [77], and occur more frequently in larger tumours [50], as necrotic components fail to reabsorb. Following intravenous contrast administration, SPT shows a heterogeneous nature in the arterial phase, even when small in size, followed by a progressive enhancement in the portal venous phase [78].

Surgical resection may be considered without prior biopsy if the presentation is classic. In atypical presentations, diagnosis is achieved through histological examination following biopsy.

Tumour resection is the treatment with curative intent, with a success rate close to 90% [79]. SPTs usually have a benign behaviour but malignancy has been reported in 10–15% [49,68,70]. Even if metastatic, the prognosis is good, as long an R0 resection is achieved [80]. The only significant proven malignancy predictors are pancreatic duct dilatation, vessel encasement and the presence of metastases [81]. It is extremely important to continue surveillance in the long term, as SPTs are prone to recur and develop metastases as a late event, even years after surgery [82].

**Figure 4:**
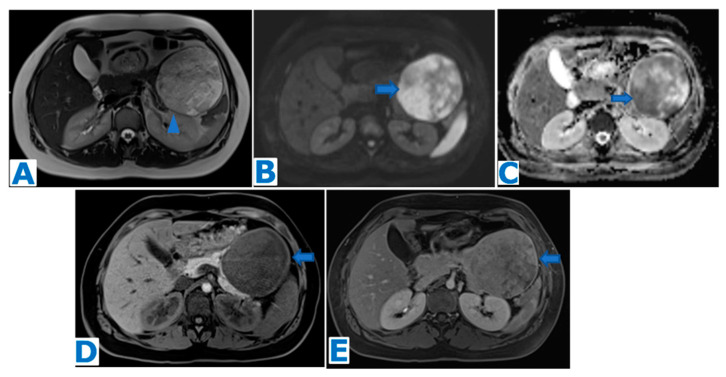
(**A**): axial T2WI, (**B**): DWI, (**C**): ADC, (**D**): axial NE FST1WI, (**E**): axial CE portal venous phase FST1WI. Pancreatic solid pseudopapillary tumour in a 20-year-old woman as an incidental finding during a pregnancy check-up**.** A 10 cm pancreatic mass was found, with a fibrous capsule (arrowhead in (**A**)), diffusion restriction (arrows in (**B**,**C**)) and heterogeneous enhancement (arrow in (**D**,**E**)). Imaging findings were compatible with a pancreatic SPT, and it was histologically proven following distal pancreatectomy.

**Figure 5:**
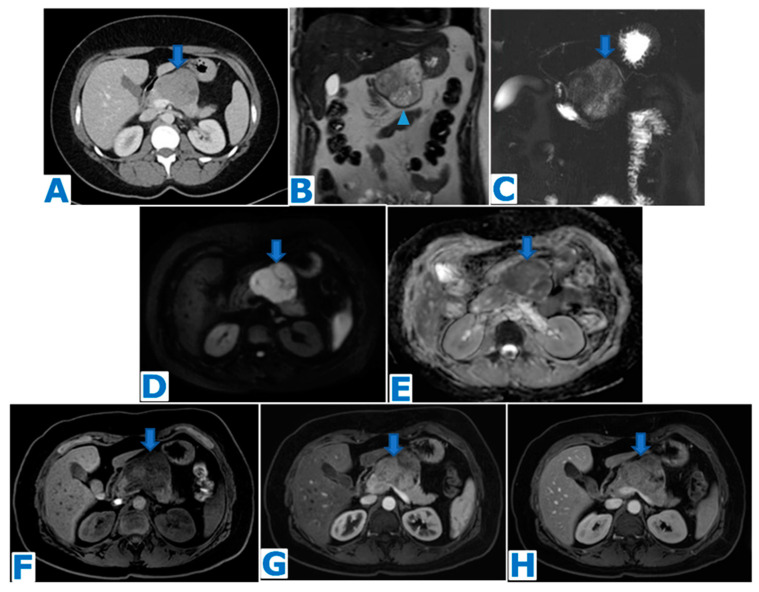
(**A**): axial CE portal venous phase CT, (**B**): coronal T2WI, (**C**): MRCP, (**D**): DWI, (**E**): ADC, (**F**): axial NCE FST1WI, (**G**): axial CE pancreatic parenchymal phase FST1WI, (**H**): axial CE portal venous phase FST1WI. Pancreatic solid pseudopapillary tumour in a 30-year-old woman as an incidental finding during a CT scan for abdominal pain. CT showed a mass within the body of the pancreas (arrow in (**A**))**.** The lesion was well defined by a fibrous capsule (arrowhead in (**B**)). MPD was displaced, with normal caliber (arrow in (**C**)). The lesion showed diffusion restriction (arrow in (**D**,**E**)) and progressive heterogeneous enhancement (arrow in (**F**–**H**)). Due to the microcystic appearance in T2WIs, the lesion was initially thought to be a microcystic serous cystadenoma, even if it lacked some characteristic features. Nevertheless, given its size and presence of symptoms, it was removed. Histological examination revealed a SPT.

### 3.2. Pancreatic Schwannoma (Figure 6)

Pancreatic schwannoma (PS) is a rare tumour that arises from Schwann cells found in the sheath of vagus nerve branches on their course through the pancreas [83].

Only 10% of cases are associated with genetic disorders, such as neurofibromatosis type 2 (NF2), multiple meningiomas and schwannomatosis, and, rarely, with neurofibromatosis type 1 (NF1), with an increased risk of malignant transformation [84].

There are less than 80 reported cases in the literature, with most of the cases occurring in adults (average age 55 years), with a slightly higher incidence in women [85].

Patients mostly present with non-specific abdominal complaints [86], although the prevalence of symptoms suspicious for a PDAC (such as weight loss, palpable mass and jaundice) is not neglectable [85].

Levels of CA 19-9 and carcinoembryonic antigen (CEA) are usually within normal values.

Most tumours have been found within the head [87].

Tumour size varies greatly and, with increasing size, there is also proportionate likelihood of degeneration occurring. Microscopically, two distinct areas are found within the tumour: Antoni A, solid, with a compact cellular organisation and a well-developed vascular net, and Antoni B, hypocellular with loose myxoid stroma, less vascularity and degenerative alterations (haemorrhage, calcification, cyst formation, hyalinisation and xanthoma infiltration) [88,89]. The tumour’s appearance is determined by the proportion of Antoni A and B areas; thus, the imaging features are non-specific and preoperative diagnosis is challenging [90].

On CT, benign schwannomas are usually depicted as encapsulated round masses with a variable proportion of avidly enhancing (Antoni A areas) and non-enhancing (Antoni B areas) components, following intravenous contrast administration [91]. On MR, hypointense signal in T1WIs and heterogeneously hyperintense signal in T2WIs are commonly found [92], in addition to progressive enhancement in T1WIs [93].

Suspicious signs of malignancy are rapid growth, invasion of neighbouring structures, a solid inhomogeneous and irregular mass with avid contrast enhancement and associated thrombosis [94].

PS is usually associated with a hypermetabolic appearance on FDG-PET, even if benign [95].

Differential diagnosis should include SPT, pNEN and pancreatic cystadenoma. The diagnosis of PS should be considered when a well-circumscribed lesion with or without a cystic component is encountered, showing increased FDG uptake on PET-CT [95].

Diagnosis is achieved after histological examination following EUS-guided biopsy.

If asymptomatic, a conservative management may be considered, given its benign nature and stable size or slow growth rate [96]. On the other hand, if symptomatic, resection should be considered. Follow-up after surgery should be carried on, as the risk of recurrence remains unknown [97].

**Figure 6:**
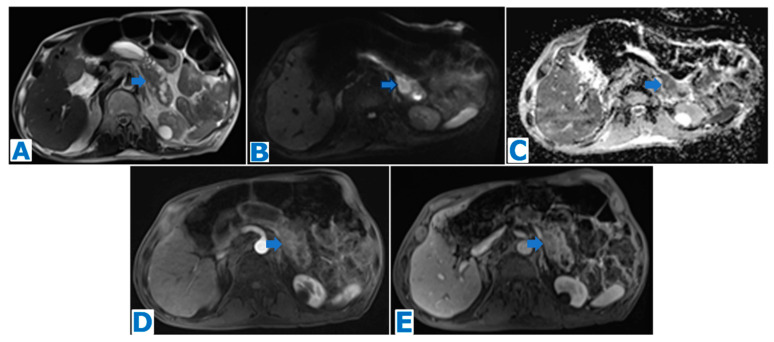
(**A**): axial T2WI, (**B**): DWI, (**C**): ADC, (**D**): axial CE early arterial phase FST1WI, (**E**): axial CE pancreatic parenchymal phase FST1WI. Pancreatic schwannoma in a 70-year-old male patient during follow-up for a duodenal gastrointestinal stromal tumour (GIST), removed five years prior through cephalic duodenopancreatectomy. A pancreatic lesion is noted within the pancreatic tail (arrow in (**A**)). Observe MPD dilatation unrelated to the lesion, due to surgical procedure. The lesion showed diffusion restriction (arrow in (**B**,**C**)) and progressive heterogeneous enhancement following intravenous contrast administration (arrow in (**D**,**E**)). EUS-guided FNA obtained fragments of mesenchymal tissue with minimal nuclear atypia and positive immunostaining for anti-S-100, and cytological report concluded schwannoma. Tumour board decided conservative management and the lesion is currently under surveillance.

### 3.3. Purely Intraductal Pancreatic Neuroendocrine Tumour (Figure 7)

Intraductal growth of a pNEN is encountered in two different scenarios. Most frequently, it is found in the form of a parenchymal lesion that extends into the pancreatic duct and grows along its extent. This presentation is very rare and very few cases have been published [98,99,100,101,102,103,104]. The other, and even rarer, setting is a true intraductal origin, where a NEN arises within the main pancreatic duct as a polypoid mass that grows along the duct [98,105,106] but it is not connected to a parenchymal lesion [107]. Only seven cases of purely intraductal pNENs have been reported in the literature [107]. Purely intraductal pNEN has been hypothesised to rise from totipotential stem cells located within the epithelium of the main duct [108]. As the tumour grows, the tumour may block the duct lumen and, as a result, it can cause pancreatitis. In fact, these tumours frequently present as a chronic pancreatitis. This exclusively intraductal lesion is not conspicuous on CT, and it may be obscured by the pancreatitis signs, so it is most frequently diagnosed after surgery. MRCP proves to be very useful as it can depict the intraductal tumour as a filling defect. Intraductal pNEN may also be identified following intravenous contrast administration as an avidly enhancing lesion in the arterial phase. This type of presentation occurs mostly associated with non-functioning pNENs [107]. An inflammatory stricture in the setting of chronic pancreatitis constitutes the other differential diagnosis possibility. There are so few cases in the literature that no data can be extrapolated.

**Figure 7:**
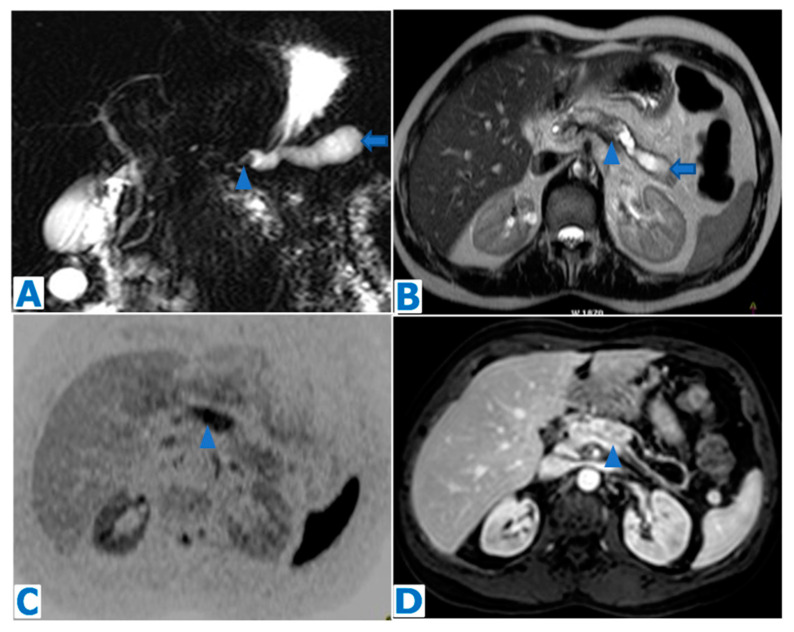
(**A**): MRCP, (**B**): axial T2WI, (**C**): DWI, (**D**): axial CE arterial phase FST1WI. Intraductal pancreatic NEN in a 55-year-old patient with a known history of a testicular tumour, admitted for acute pancreatitis, with no risk factors. Note the marked dilatation of the distal MPD (arrows in (**A**,**B**)) with a proximal filling defect (arrowheads in (**A**,**B**)) which corresponds to the intraductal tumour. The intraductal mass shows diffusion restriction (arrowhead in (**C**)) and intense enhancement following intravenous contrast administration (arrowhead in (**D**)). A total pancreatectomy was decided by the MDT and histological examination concluded grade 2 intraductal pNEN.

### 3.4. Pancreatic Solitary Fibrous Tumour (Figure 8)

This type of extrapleural solitary fibrous tumour is a fibroblastic mesenchymal tumour, previously known as haemangiopericytoma. It was first described in the pleura in 1931 [109], derived from mesenchymal cells from pleural connective tissue, but since then, it has been documented in almost every anatomic site, including the retroperitoneum [110].

Pancreatic solitary fibrous tumour (PSFT) is a rare neoplasm, with only 29 cases reported [111]. It shows no gender preference, and the median age reported at diagnosis is 53 years [112]. The main symptoms reported at presentation are abdominal pain and jaundice, though most frequently tumours are incidental findings [113].

Patients may present with refractory and recurrent hypoglucemia as a paraneoplastic syndrome (Doege–Potter syndrome), caused by an increased production of insulin-like growth factor II [113]. Being a mesenchymal tumour, there is no association with tumour markers.

PSFT arises most commonly within the pancreatic head [111].

It shows a true capsule and well-defined margins, and it does not tend to invade the surrounding parenchyma [114]. Its most salient feature is its hypervascularity, and it usually enhances homogeneously and progressively in the arterial and portal phase [115]. In larger tumours, central necrosis occurs, and it has been described that a malignant type may present a heterogeneous appearance with haemorrhage, necrosis and calcifications [115].

Dilatation of the main pancreatic duct has been observed in some cases, as well as biliary dilatation in tumours located within the head [116], but these findings are not a constant, despite the large size of tumours. Lymphadenopathies are not frequently associated [111].

FDG-PET has not been shown to be useful in distinguishing indolent from aggressive PSFT [117], contrary to previous hypotheses.

The main differential diagnosis based on imaging findings is pNEN [118]. Other options should include leiomyosarcoma, GIST, perivascular epithelioid cell tumour (PEComa) and SPT in younger patients.

Definite diagnosis is achieved by EUS-guided biopsy. Curative treatment is complete surgical resection [119], with good results, since most of the published cases were disease free after surgery [115]. Adjuvant radio- or chemotherapy treatments have not achieved successful results [120]. Negative margins have proved to decrease the rate of local recurrence and to improve survival [120]. Follow-up is recommended, as about 12–22% of all solitary fibrous tumours are aggressive, with local recurrence and metastases [121].

**Figure 8:**
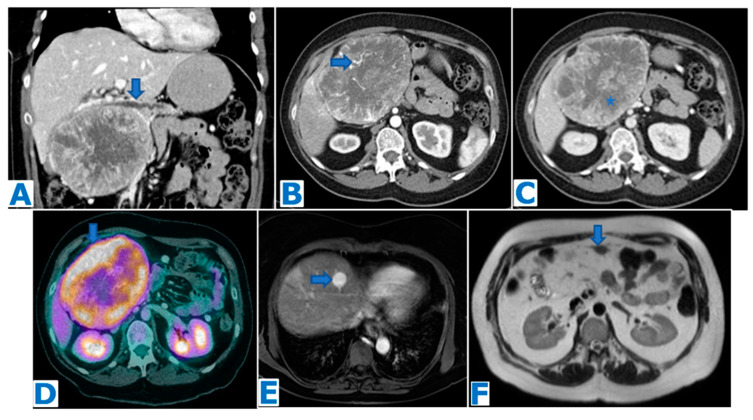
(**A**): CE-CT portal phase coronal MPR, (**B**): axial CE arterial phase CT, (**C**): axial CE portal venous phase CT, (**D**): FDG-PET CT. Follow-up images (**E**): axial CE arterial phase FST1WI, (**F**): axial T2WI. Malignant pancreatic solitary fibrous tumour in a 47-year-old patient who presented with a palpable mass within the right hypochondrium. She had a history of a nasal fibrous solitary tumour 10 years prior. CT showed an enormous solid mass in the head of the pancreas, causing mild dilatation of the pancreatic duct (arrow in (**A**)). Note the central necrosis (* in (**C**)) and the hypervascularity of the non-necrotic periphery (arrow in (**B**)), which is highly metabolic on the FDG-PET (arrow in (**D**)). The patient underwent a total pancreatectomy and the histological examination concluded PSFT. It turned out to have a malignant outcome and the patient developed liver (arrow in (**E**)) and omental (arrow in (**F**)) metastases within the year following the surgery.

## 4. Malignant Lesions (Table S3)

### 4.1. Acinar Cell Carcinoma (Figure 9 and Figure 10)

Acinar cell carcinoma is a rare epithelial malignant primary pancreatic tumour, named after the acinar differentiation of its cells. Even though acinar cells constitute most of the pancreatic parenchyma, acinar cell carcinoma (ACC) paradoxically represents less than 2% of primary pancreatic neoplasms [122].

ACC occurs mostly in men (men to women ratio of 3.6) with a bimodal presentation, with two incidence peaks at 8–15 and 60 years [123,124,125].

It arises throughout the pancreas, with no favoured location.

Presenting symptoms are non-specific, with abdominal pain and weight loss being the most common. Pancreatitis and obstructive jaundice are rare [126,127] as, despite their large size, ACCs do not tend to cause ductal obstruction [128,129].

Elevated lipase, secreted by the tumour, may be the presenting sign of ACC and may be used as a tumour marker [130]. As a result, fat necrosis may be triggered, either subcutaneously, presenting as nodules, or within the cancellous bone, causing polyarthralgia [131,132]. These symptoms, together with peripheral eosinophilia, constitute a paraneoplastic syndrome [133] that may occur after tumour recurrence. An elevated alpha-fetoprotein may sometimes be found [134]. Levels of CA 19-9 and carcinoembryonic antigen (CEA) are usually within normal values.

At the time of presentation, almost half of patients present with hepatic and lymph node metastases [135].

On cross-sectional imaging, ACC usually appears as a large (average size at diagnosis of 10cm [136,137,138]), well-defined and oval or round exophytic mass (it may even be found attached to the surface of the pancreas on the histological examination [130]). Calcifications are found in one third of patients [136,137,139]. It usually shows a solid appearance, but internal haemorrhage, necrosis and cystic changes are common in larger lesions [136]. On unenhanced CT, it is usually iso-hypodense to the pancreatic parenchyma, and it shows a hypovascular nature, hypoenhancing in the arterial phase and becoming more enhancing than the pancreatic parenchyma in the portal venous phase [140]. An enhancing capsule may also be identified.

Concerning the cross-sectional imaging test of choice, the combination of CT and MR works well in depicting the imaging features. MR outperforms CT in describing tumour limits, intratumoral bleeding, local invasion and ductal dilatation, whereas CT is better at detecting calcification [141].

Differential diagnosis should include PDAC, pNENs, SPT and, in children, also pancreatoblastoma. PDAC usually shows a smaller size with no calcification or cystic changes [142]. Its margins are not well delineated, and invasion of neighbouring structures is one of its hallmarks. ACCs are often mistaken for large pNENs, as they may show heterogeneous density/SI due to haemorrhage, necrosis, cystic changes and calcifications, but ACCs are mainly hypovascular. SPTs may also mimic ACCs, but the target population is the key: they occur almost exclusively in young women, in which ACCs rarely occur [143]. Pancreatoblastoma may cause a differential diagnosis issue, as it usually occurs in infants and children [144]. Its frequently also presents with liver metastases, but it is more aggressive than ACC.

Even if almost half of patients present at diagnosis with hepatic and regional lymph nodes metastases [135,145], ACC shows a better prognosis than PDAC, with a 5-year survival rate of 50% [146,147]. However, ACC has a higher rate of recurrence [141].

Surgical resection with negative margins is the only therapeutic approach that improves long-term survival. Recent studies suggest that the outcome of combining surgery and chemotherapy is more favourable than that of only surgery [148].

**Figure 9:**
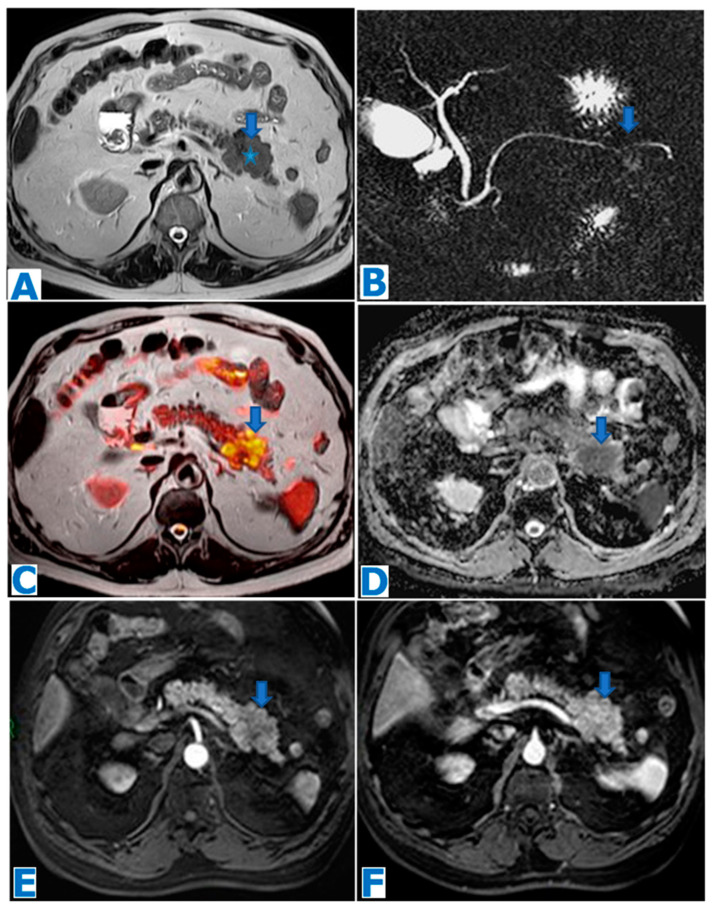
(**A**): axial T2WI, (**B**): MRCP following secretin injection, (**C**): axial T2 DWI fusion, (**D**): ADC, (**E**): axial CE pancreatic parenchymal phase FST1WI, (**F**): axial CE portal venous phase FST1WI. Acinar cell adenocarcinoma in a 79-year-old patient with previous episodes of pancreatitis of unknown cause and elevated lipase in current laboratory results. A solid well-defined mass (arrow in (**A**)) with lobulated contours and minimal MPD stenosis (* in (**A**)) is found in the distal pancreas. It shows diffusion restriction (arrows in (**C**,**D**)). Note the duct penetrating sign following secretin injection (arrow in (**B**)). It is hypoenhancing in the early arterial phase (arrow in (**E**)) with progressive enhancement during pancreatic parenchymal phase (arrow in (**F**)). No adenopathies are found. Findings were non-specific and did not fulfill the diagnosis criteria for PDAC. Diagnosis was achieved at histological examination following EUS-guided FNB.

**Figure 10:**
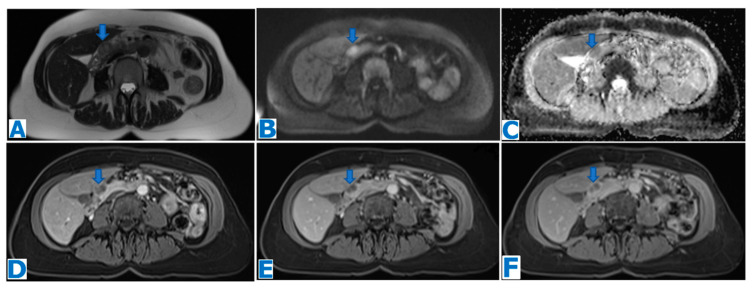
(**A**): axial T2WI, (**B**): DWI, (**C**): ADC, (**D**): CE pancreatic parenchymal phase FST1WI, (**E**): axial CE portal venous phase FST1WI, (**F**): axial CE delayed venous phase FST1WI. Acinar cell adenocarcinoma incidentally discovered in a 70-year-old female patient during a routine echography. Note the lesion within the head of the pancreas, rather exophytic and heterogeneous (arrow in (**A**)), with diffusion restriction (arrows in (**B**,**C**)). During the dynamic sequences following intravenous contrast administration (arrows in (**D**–**F**)), the lesion shows capsular enhancement while the center remains hypointense, due to necrosis/cystic changes. Due to its exophytic appearance, the lesion was thought to be within the pancreaticoduodenal groove and, hence, was diagnosed as a GIST. Histopathological examination following EUS-guided FNB revealed an ACC.

### 4.2. Undifferentiated Carcinoma with Osteoclastic-like Giant Cells (Figure 11) 

Undifferentiated carcinoma with osteoclastic-like giant cells (UCOGC) is an extremely rare and aggressive subtype of pancreatic adenocarcinoma. It constitutes less than 1% of all pancreatic malignant tumours [149].

Its histogenesis is not clear, as at the time of diagnosis, it presents with a large size and its relation to the pancreatic duct is difficult to establish. About 20% of cases seem to arise from mucinous or intraductal papillary mucinous neoplasms (IPMNs) [150] and it has been hypothesised that it has an epithelial origin with a mesenchymal transition [150,151]. The epithelial to mesenchymal transition is a transient and reversible transformation which is normally activated during embryonic development and tissue repair but also during carcinogenesis [152,153]. Through this step, tumoral cells acquire mesenchymal features that enable them to invade adjacent vessels and distant organs [154].

Two phenotypes have been described [155], a pure form containing only osteoclast-like giant cells, with a better prognosis than the mixed form, a combination of undifferentiated carcinoma of the pancreas and osteoclast-like giant cells forming a very aggressive tumour with a poor outcome. This mixed form constitutes a distinct variant from undifferentiated carcinoma of the pancreas [156]. UCOGC may occur in association with PDAC [157].

UCOGC occurs more commonly in women (women:men ratio of 13:8) with higher prevalence in middle-aged and elderly patients [158].

Presenting symptoms are non-specific and consist of upper abdominal pain, weight loss and/or anorexia. Jaundice and steatorrhoea have been described in 25% of cases [159].

CA 19-9 and CEA serum levels have been reported to be increased in some patients [160].

Favoured locations are the body and tail of the pancreas [157].

Biliary ducts and pancreatic duct dilatation may occur [151,158,159], as UCOGC seems prone to grow intraductally [151].

At presentation, UCOGCs are usually large lesions [161], locally aggressive, with a tendency to invade adjacent structures. Lymph node involvement and distant metastases are rarely encountered [151,161].

On cross-sectional imaging, appearance may vary and it displays non-specific features, either hypovascular [158] or hypervascular [162]. Hypervascular behaviour may be explained by a relationship to giant cell tumours of the bone, also hypervascular, so enhancement is proportionate to the volume of the osteoclastic cell component [151]. Haemorrhage [163], cystic changes [162], necrosis [158] calcification [164] and vascular invasion may occur [163].

UCOGC may be misdiagnosed as PDAC, mucinous carcinoma [165], SPT [158], pNEN [166] and pancreatic pseudocyst [167].

Diagnosis follows histological examination after EUS biopsy. Surgical resection is the treatment of choice. The efficacy of chemotherapy and radiotherapy has not been proved yet.

Its prognosis is variable, ranging from a few months to up to ten years as reported in the literature [168]. It was traditionally considered worse than that of PDAC [169,170] due to the advanced stage at diagnosis [165] and its tendency to recur even after complete resection [165,171].

Another analysis result of another series concluded that the prognosis (5-year survival >50%) is considerably better than that of PDAC [150]. It has been hypothesised that these discordant prognosis results are probably due to the use of wrong terminology [172] and it is clear that true UCOGCs have a more indolent behaviour [164], especially the pure form [173].

The underlying reasons for the better prognosis compared to PDAC may be its slower local spread, more indolent nature, better response to surgery and/or chemotherapy, less nodal involvement and fewer distant metastases [174].

The most important criterion for prognosis is the presence of an associated PDAC [173].

**Figure 11:**
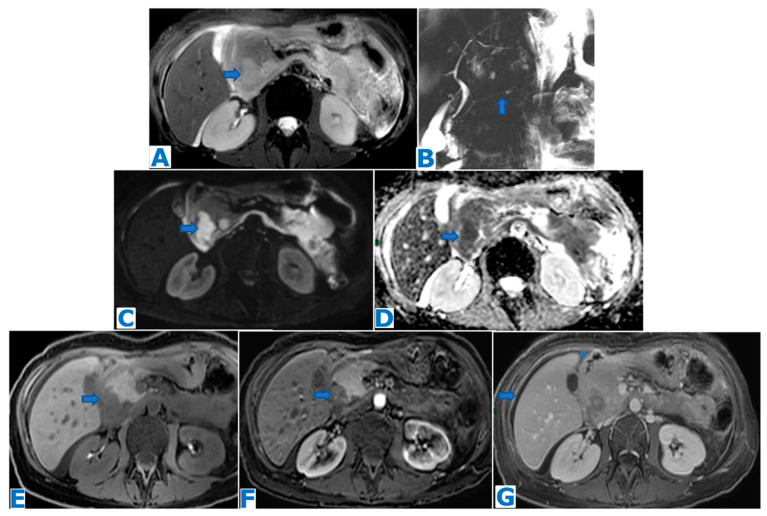
(**A**): axial FST2WI, (**B**): MRCP, (**C**): DWI, (**D**): ADC, (**E**): NEC axial FST1WI, (**F**): axial CE pancreatic parenchymal phase FST1WI, (**G**): axial CE portal venous phase FST1WI. UCOGC in a 45-year-old patient who presented with bloating. A cephalic pancreatic mass was identified, hyperintense in T2WIs (arrow in (**A**)), with MPD integrity (arrow in (**B**)) and no biliary duct dilatation, diffusion restriction (arrows in (**C**,**D**)) and scarce progressive enhancement in the dynamic sequences (arrows in (**E**–**G**)). These non-specific features did not fulfil PDAC diagnostic criteria. Ascites (* in (**G**)) and peritoneal deposits (arrow in (**G**)) were also found. Histology examination following EUS-guided FNB revealed a UCOGC.

### 4.3. Pancreatic Adenosquamous Carcinoma (Figure 12)

Pancreatic adenosquamous carcinoma (PASC) is a rare and aggressive variant of PDAC which is frequently misdiagnosed as such on imaging or even histopathologically. Its actual prevalence is thus inexact and has been reported to range from 0.38 to 10% [175,176,177]. A squamous cell component of at least 30% among glandular elements of PDAC has been a requisite for the diagnosis [178,179], although the required percentage recently has been questioned, as the proportion of squamous carcinoma does not have a clinical correlation and its evaluation remains subjective [180,181,182,183].

As squamous cells are not found in normal pancreatic tissue, the pathophysiology remains a mystery. Three hypotheses have been reported. The leading theory proposes that since squamous cells are found in the setting of chronic pancreatitis or in the event of tumour ductal obstruction and these conditions are associated with PDAC, squamous carcinoma could arise from a preexisting adenocarcinoma, through metaplastic changes [179,181,184,185]. PASC could also be the result of two different neoplastic pancreatic cell lines merging [184,186,187] or even having a common origin, as the third theory implies, where certain pluripotential primitive cells would differentiate into adenocarcinoma and others into squamous cell carcinoma, resulting in a tumour with both cell types [179,184].

Squamous carcinoma tends to show intercellular bridges and/or focal keratin pearl formation within its cells. However, PASC frequently presents as a poorly differentiated tumour and the use of immunochemistry is often needed to confirm the differentiation [188].

Elevated levels of CA 19-9 and CEA are found in most patients [189]. Hypercalcemia of malignancy is found in some cases, probably related to high serum levels of parathyroid hormone-related protein [190,191,192].

There is a higher prevalence in men and average age at presentation is 68 years [175].

Presenting symptoms are non-specific and indistinguishable from those of PDAC (abdominal pain, weight loss, anorexia and jaundice) [186,193].

Most frequently, at presentation, PASC is locally advanced or has distant metastases [194]: liver, lung [195,196] and even bone and skin [197,198,199].

Like PDAC, the head of the pancreas is the most common location but it arises within the body–tail more often than PDAC [175].

It is frequently associated with MPD dilatation and CBD dilatation when found within the head.

PASC tends to be larger than PDAC. It appears as a round lobulated mass with extensive central necrosis which causes an hyperintensity in T2WIs greater than that of PDAC [200] and a fibrous capsule that enhances progressively. Enhancement is overall considered to be greater than that of PDAC [201]. Another presenting imaging feature which may be helpful to distinguish it from PDAC is the frequently associated portal vein tumour thrombus [189,201,202,203].

Diagnosis may be achieved presurgically through an EUS-guided biopsy.

Complete resection is the only potentially curative treatment, although only 15–20% of patients are surgical candidates. A less favourable outcome has traditionally been associated with PASC, compared to PDAC, with a worse survival in patients who have undergone resection [204]. However, surgical resection has been shown to significantly improve median patient survival: median overall survival after surgery is 12 months, while in PDAC it is 16 months [175]. On the other end of the differentiation spectrum, squamous cell carcinoma appears to be an even more aggressive tumour, with worse survival data, which might suggest that the squamous element is a worsening prognosis factor [204].

**Figure 12:**
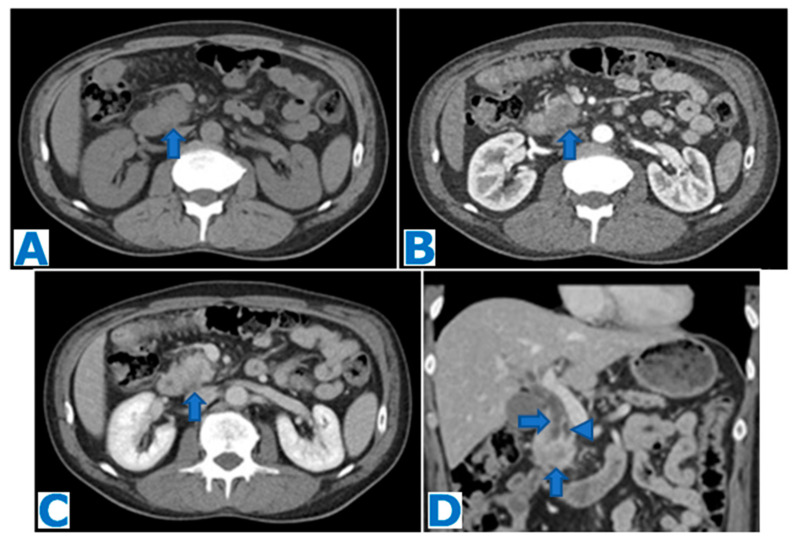
(**A**): axial NCE-CT, (**B**): axial CE pancreatic parenchymal phase CT, (**C**): axial CE portal venous phase CT, (**D**): CE portal phase CT coronal MPR. Adenosquamous carcinoma. A 60-year-old patient with elevated liver enzymes was discovered to have a pancreatic mass during an ultrasound. Note the subtle contour abnormalities of the pancreatic head on the unenhanced CT (arrow in (**A**)) and the progressively enhancing lesion (arrow in (**B**,**C**)). Observe the biliary duct (arrow in (**D**)) and the upstream MPD dilatation (arrowhead in (**D**)). EUS-guided FNA concluded moderately differentiated adenocarcinoma and, since the tumour was resectable, the patient underwent surgery. Histological examination proved it to be a PASC. Retrospectively, it shows a greater enhancement than a typical PDAC.

### 4.4. Colloid Carcinoma (Figure 13)

Colloid carcinoma (CC) of the pancreas, alternatively referred to as mucinous non-cystic carcinoma, is a rare variant of ductal adenocarcinoma, which occurs with a rate of 1% of all pancreatic tumours [205,206].

Its hallmark is the abundant presence of extracellular mucin (adding up to at least 50% of the tumour), with malignant cells floating within it [207]. This mucinous component is the reason why it was previously categorised as mucinous cystadenoma or signet-ring cell carcinoma of the pancreas [206].

It appears that there is a slightly higher prevalence in men [205,208,209] and age at presentation ranges within the seventh decade [205].

Tumour markers (including CEA and CA 19-9) are usually elevated [205].

Presenting symptoms resemble those associated with PDAC: abdominal pain, jaundice and weight loss [179]. Almost half of patients with CC present a history of pancreatitis [209].

Most colloid carcinomas are associated with intestinal-type invasive IPMN although they may also arise de novo [210,211], and these types occur most frequently within the head of the pancreas [211,212]. Another less frequent association has been described with mucinous cystic tumours, involving preferably the tail of the pancreas [205].

CC is a slow-growing tumour that shows local invasion rather than disseminated disease [212]. Lymph node metastases and vascular invasion occur less frequently in CC than in PDAC [213,214].

The presence of dilatation of the main pancreatic duct will depend on whether the CC derives from an IPMN; if so, the tumour will be intraluminal, either the main or branch duct, and there will be downstream MPD dilatation [213]. If the tumour is unrelated to an IPMN, no dilatation will be found. Bile duct dilatation may occur in tumours arising from the head of the pancreas.

The reported tumour size at presentation ranges from 1 to 16cm [205,206]. Usually, they present on CT with a lobulated appearance and slightly ill-defined margins [215]. Calcifications are often found [215]. In T2WIs, CC shows very bright signal intensity with internal septa and a salt and pepper appearance, these features being consistent with the abundant mucin lakes with floating stroma and tumour cells [210]. Enhancement will happen typically progressively so at a delayed phase it will be more conspicuous. Enhancement will be observed internally in a sponge-like fashion, due to the enhancing stroma amidst the mucin lakes, which will enhance poorly and peripherally, associated with induced desmoplastic reaction [210,215].

CC has an indolent behaviour, and its prognosis is superior to PDAC: 5-year survival rates of 40–60% vs. 10–15%, respectively [216,217]. One of the reasons explaining this better prognosis is the mucin, which surrounds the cells and acts as a barrier preventing their spread [207,216]. The other reason lies within the surface glycoproteins present in colloid carcinoma: MUC1 is present in PDAC on the luminal aspect or throughout the cells, whereas CC expresses MUC1 on the basal surface [218]. Also, another surface glycoprotein found in CC, MUC2, not found in PDAC, has been described to have tumour suppressor activity [215].

A misleading cystic appearance due to the abundant mucin production may cause a misdiagnosis of cystic tumours, such as IPMN or a mucinous cystic adenocarcinoma [219]. Hallmarks to distinguish IPMN-unrelated colloid carcinomas from IPMN in cross-sectional images are an absence of communication with the MPD and of intraductal papillary components and a lack of downstream pancreatic ductal dilatation, features that can be successfully assessed with MRCP. Also, the typical papillary bulging into the duodenal lumen and spillage of mucin from the ampulla of Vater, typical findings on endoscopic retrograde cholangiopancreatography (ERCP), will not be present in CC [210,220]. Mucinous cystic adenocarcinomas, on the other hand, are large well-defined unilocular or macrocystic lesions with enhancing soft tissue components, different from the not-so-well-defined CC with progressive internal enhancement, besides the fact that the target populations are women.

In the event of an intraluminal CC communicating with the MPD, features will be difficult to distinguish from invasive IPMN on cross-sectional imaging and ERCP will be essential to rule IPMN out.

Even though FNA is useful to describe the large amounts of mucin, it may not provide enough data to complete the diagnosis [221]. The presence of malignant epithelial cells within a mucin magma should provide definitive diagnosis [179]. However, given the rarity of this entity, CCs are typically diagnosed during histological examination following surgery.

Surgery is recommended as the only curative treatment in eligible patients [221]. A recent study has suggested that adjuvant chemotherapy may not be effective for CC [222]. The survival rate has been reported to be better than for PDAC (5-year survival rate of 57%) [223]. Long-term surveillance is recommended to detect recurrence [221].

**Figure 13:**
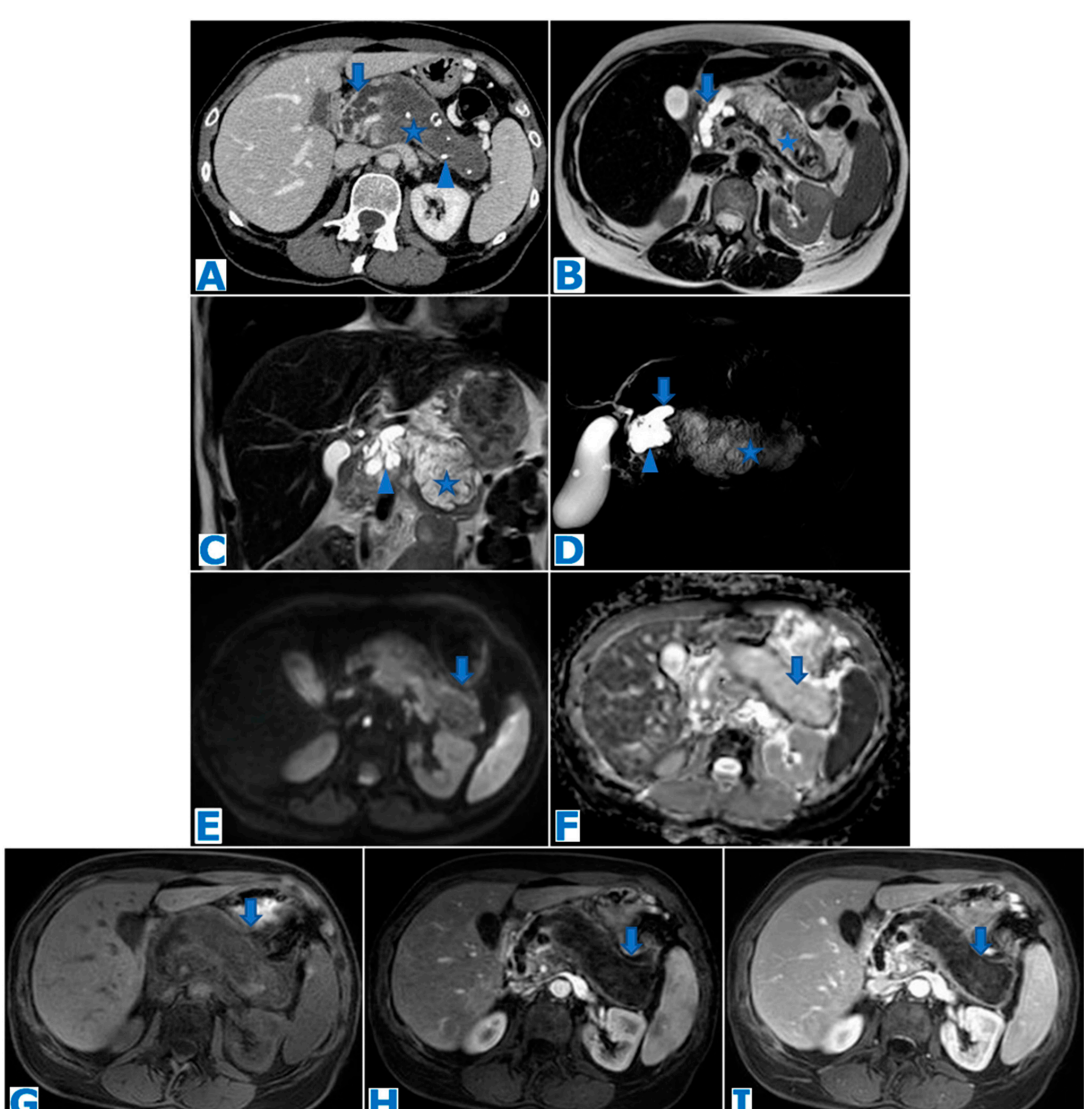
(**A**): axial CE portal venous phase CT, (**B**): axial T2WI, (**C**): coronal T2WI, (**D**)*:* MRCP, (**E**): DWI, (**F**): ADC, (**G**): axial NCE FST1WI, (**H**): CE pancreatic parenchymal phase FST1WI, (**I**): axial CE portal venous phase FST1WI. Colloid carcinoma. Patient is a 52-year-old woman with left upper quadrant pain for the previous six months and weight loss (15 kg). Blood laboratory tests are anodyne. Observe the intraluminal mass within the body and distal pancreas (* in (**A**–**D**)), notice its salt and pepper pattern in T2WIs (* in (**B**,**C**)) and how it is partly calcified (arrowhead in (**A**)). The lesion is associated with downstream MPD dilatation (arrow in (**A**,**B**,**D**)) and side branch ecstasy (arrowhead in (**C**,**D**)). There is no diffusion restriction (arrow in (**E**,**F**)). In the dynamic sequences following intravenous contrast administration, the tumour shows gradual enhancement of the periphery and the subtle septa (arrows in (**G**–**I**)). Patient underwent a cephalic duodenopancreatectomy and diagnosis was pathologically proven.

### 4.5. Primary Pancreatic Leiomyosarcoma (Figure 14 and Figure 15)

Primary pancreatic leiomyosarcoma (PPLM) belongs to the group of malignant mesenchymal tumours that may originate in the pancreas, along with malignant peripheral nerve sheath tumours, undifferentiated pleomorphic sarcomas, liposarcomas, rhabdomyosarcomas, solitary fibrous tumour and primitive neuroectodermal tumours (PNETs), among which it ranks first in frequency [224]. It is a very rare and aggressive tumour, which accounts only for 0.1% of malignant pancreatic neoplasms [225].

Its cells show smooth muscle features [226], a fact that has given rise to theories regarding the walls of intrapancreatic vessels or the smooth muscle cells of the pancreatic ducts as possible origins [226]. These theories may be the rationale behind the close relationship between the tumour and the vessels/duct [226,227,228,229].

It occurs most frequently during the fifth decade; gender predominance is not clear [226,230,231,232]. An association with East Asian ethnicity has been recently proposed, with a higher prevalence of regional invasion [233].

Presenting symptoms are non-specific and variable, and the most frequently encountered complaints are abdominal pain/tenderness, weight loss and a palpable abdominal mass [229].

Since it is a mesenchymal tumour, there is no association with tumour markers.

No preferred site within the pancreas has been described [226], and there is similar incidence between the head and the body–tail [232].

It is locally very aggressive and, since it is usually discovered at a late stage, invasion of neighbouring organs and vessels is a frequent feature. It is prone to metastasise to the liver, and lung metastases are also frequently present at diagnosis [232,234]. However, lymphatic spread is rare [226,229], a fact that could be helpful for differential diagnosis.

PPLMs have been described in the literature as non-specific masses on CT/MR, with size ranging from 3–25cm [230], that, as volume increases, become heterogeneous, with haemorrhagic, necrotic and cystic components, due to degenerative changes [232,235]. Peripheric enhancement is present with a large central non-enhancing component [235,236]. These features may lead to misdiagnosis of a large leiomyosarcoma as a pseudocyst [229] or a cystoadenocarcinoma [237].

Usually, there is no associated MPD dilatation. However, tumours arising from smooth cells of the pancreatic duct have been described [238].

It has been proposed that diagnosis should be entertained when confronted with a mass that fulfils the following criteria: large size, increased enhancement and absence of biliary duct dilatation [236] and other authors have added the presence of cystic/necrotic components to the list [239,240].

Differential diagnosis includes the far more frequent PDAC, and, less frequently, pNEN [240,241,242], a metastasis to the pancreas from another known primary tumour [240,243] and, more rarely, an invading leiomyosarcoma originating from adjacent organs and simulating a pancreatic primary tumour [244]. An isolated metastasis to the pancreas from a distant leiomyosarcoma is extremely rare [245], with female genital tract, gastrointestinal tract, soft tissues of the extremities and retroperitoneum as most common sites of origin [246].

Diagnosis is usually achieved after histological examinations and immunohistochemical staining [232], following surgery or intraoperative biopsy. EUS-guided FNA often comes up with false negative results due to the cystic and fibrous nature of the lesion [235,247].

In the absence of organ/vessel invasion or distant metastases, radical resection with negative margins stands as the only potentially curative treatment [248].

Radiation and chemotherapy have not achieved clinical success, as for other leiomyosarcomas [249,250,251].

It is usually associated with a poor outcome; the median survival time in a series of 49 cases [252] was 48 months.

**Figure 14:**
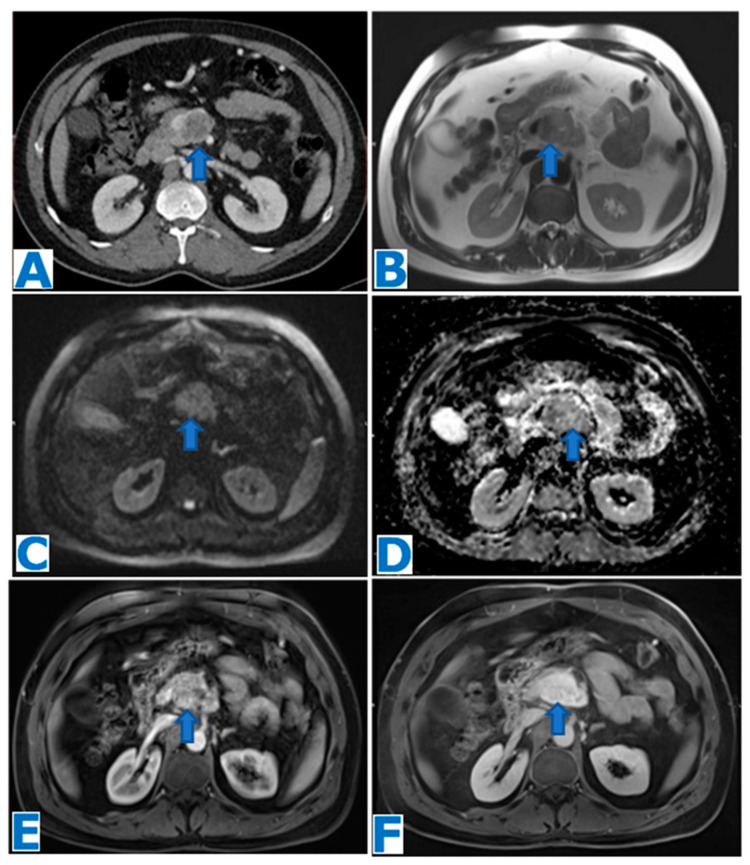
(**A**): axial CE portal venous phase CT, (**B**): axial T2WI, (**C**): DWI, (**D**): ADC, (**E**): axial CE pancreatic parenchymal phase FST1WI, (**F**): axial CE portal venous phase FST1WI. Primary pancreatic leiomyosarcoma incidentally discovered in a 53-year-old patient during a routine check-up. Note the heterogeneous mass within the pancreatic isthmus (arrow in (**B**)) with compression of the superior mesenteric and splenic vein (arrow in (**A**)). No biliary or pancreatic duct dilatation is observed. The lesion shows diffusion restriction (arrow in (**C**,**D**)) and hypervascularity, with progressive enhancement following intravenous contrast administration (arrow in (**E**,**F**)). EUS-guided FNB concluded PPLM. The patient underwent radiotherapy before surgery but then refused to be operated upon and developed hepatic and muscular metastases (not shown here). Stable disease was achieved with chemotherapy for five years, but it is currently progressing.

**Figure 15:**
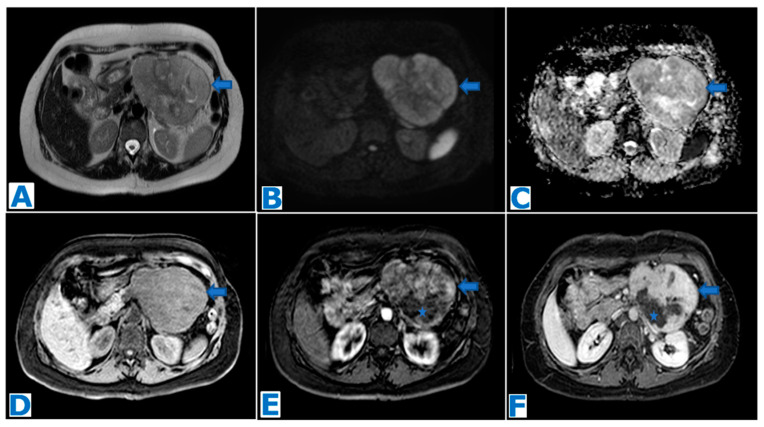
(**A**): axial T2WI, (**B**): DWI, (**C**): ADC, (**D**): axial NCE FST1WI, (**E**): axial CE pancreatic parenchymal phase FST1WI, (**F**): axial CE portal venous phase FST1WI. Primary pancreatic leiomyosarcoma incidentally discovered in a 50-year-old female patient during an ultrasound for a urinary infection. Observe the large heterogeneous mass in T2WI (arrow in (**A**)) within the distal pancreas, with diffusion restriction (arrow in (**B**,**C**)). The sequences following intravenous contrast administration show the central cystic/necrotic component (* in (**E**,**F**)) and the solid and progressively peripheral enhancement (arrow in (**D**–**F**))**.** Resectability criteria were fulfilled, and patient underwent distal pancreatectomy. Histological examination concluded PPLM.

### 4.6. Primary and Secondary Pancreatic Lymphoma (Figure 16, Figure 17 and Figure 18)

Primary pancreatic lymphoma (PPL) is an extremely rare non-epithelial tumour that accounts for less than 0.5% of all pancreatic tumours and 1% of all extranodal lymphomas [253].

PPL occurs most commonly in middle-aged patients (mean age 53 years) [254], especially Caucasians [255], and with a male prevalence [254]. It is frequently associated with immunosuppression that seems to favour the disease [256].

Patients mainly complain of abdominal pain at diagnosis. Other presenting symptoms are jaundice, which is relatively common, pancreatitis and/or gastric or duodenal obstruction [110,257]. Fever, chills, night sweats and weight loss are associated with systemic non-Hodgkin lymphoma (constituting the classic B symptoms) but are rare in PPL [254].

The most frequent subtype is B-cell non-Hodgkin lymphoma [255].

CA 19-9 usually ranges within normal limits [254], even though PPL-associated biliary dilatation may cause a mild elevation of CA 19-9 [252]; LDH is often elevated [256]. Thus, the combination of increased LDH serum levels without concurrent increased CA 19-9 should favour the diagnosis of pancreatic lymphoma [255,258].

Different morphologic patterns have been described [259], the most common is a solitary focal mass. A diffuse infiltration with pancreatic enlargement, a peripheral involvement and a multinodular type comprehend the rest of the presentations.

The focal pattern occurs mainly within the head, as the part that contains the largest concentration of lymphoid tissue [255,260]. It is depicted as a bulky, well-circumscribed mass, ranging between 2 and 14 cm [255]. It is homogeneous, and it shows progressive and delayed but limited homogeneous enhancement, to a lesser degree compared to the preserved pancreatic parenchyma [259]. Characteristically, necrosis and calcification are hardly ever present [261], although necrosis may happen secondary to concomitant acute pancreatitis, or due to a duodenal fistula causing an intratumoral collection [262]. Compared to the preserved pancreatic parenchyma, PPL is usually hypointense in T1WIs and hyperintense in T2WIs [262]. The hallmark on MR imaging is the significant diffusion restriction, similar to that of the spleen.

The infiltrative pattern leads to a diffuse, ill-defined enlargement of the pancreas and may mimic acute pancreatitis [263]. However, even if both the focal and diffuse patterns may be associated with stranding of the peripancreatic fat [264], it is minimal, unlike the marked inflammation associated with acute pancreatitis. Moreover, the typical peripancreatic collections and a concordant clinical history are absent.

Peripheral involvement occurs rarely, as a focally enlarged hypointense pancreas in T1- and T2WIs, and with a capsule-like rim, which may mimic autoimmune pancreatitis [265].

The multinodular pattern is similar to the solitary focal mass, but the lesions are smaller [254]. Differential diagnosis includes multiple metastases from hypovascular tumours and multifocal autoimmune pancreatitis.

Despite the large size previously mentioned, the main pancreatic duct is usually not dilated [254] and pancreatic atrophy is not present [264]. Nevertheless, mild pancreatic duct dilatation may still be found so its presence should not rule out the possibility of pancreatic lymphoma [261]. The biliary duct has been described for a considerable number of patients [261]. However, even if present, biliary and/or pancreatic ductal obstruction will be disproportionately milder than expected, considering the size of the mass.

As with lymphomas elsewhere, PPL may infiltrate surrounding organs, not respecting anatomic boundaries, and may displace and encase adjacent vessels but will not invade or cause stenosis or occlusion [254]. No irregularities within the vessel wall are found [257,258].

A small volume of retroperitoneal lymphadenopathy is frequently found to be associated, both peripancreatic and around the aorta and cava vein. If present below the renal veins, pancreatic ductal adenocarcinoma can be confidently excluded [259,266,267].

Secondary pancreatic lymphoma (SPL) is a direct involvement of the pancreas from peripancreatic adenopathies and, as opposed to the primary tumour, occurs more frequently, in up to 30% of lymphoma patients [257], especially in widespread nodal or extranodal disease [268]. Even in this scenario, a predominant involvement of the pancreas is quite uncommon [269]. The most common type is diffuse large B-cell non-Hodgkin lymphoma [270]. SPL may also show the different presentation patterns previously described [259]. It may be difficult to distinguish on imaging from the diffuse form of PPL, but the clinical setting is different.

PPL may be misdiagnosed as PDAC, as they share imaging features. Differentiation becomes critical as PPL is highly sensitive to chemotherapy and does not require surgery. Diagnosis is achieved after EUS-guided biopsy. Long-term regression or remission is frequently achieved, with survival rates similar to those of nodal non-Hodgkin lymphoma [255]. However, relapses occur frequently [271], especially at distant sites, like the central nervous system [255,257,272], and prolonged follow-up is recommended.

**Figure 16:**
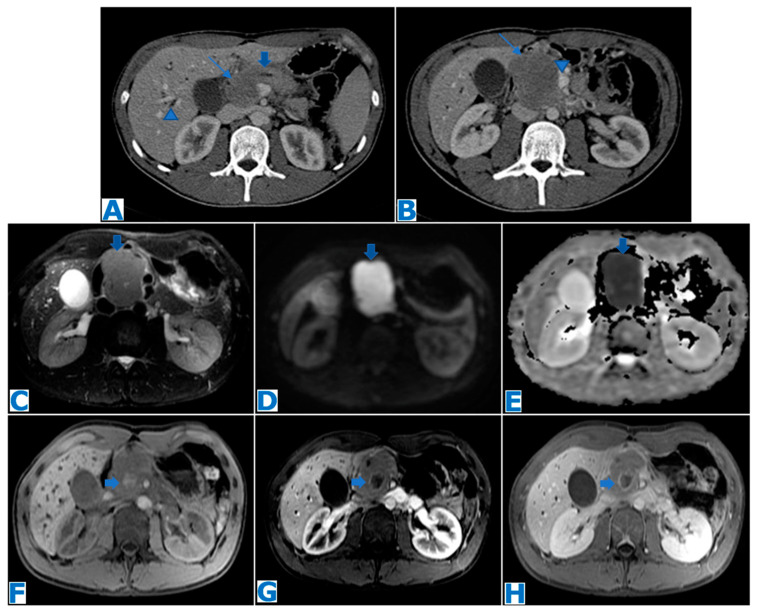
(**A**,**B**): axial CE portal venous phase CT. (**C**): axial FST2WI, (**D**): DWI, (**E**): ADC, (**F**): axial NCE FST1WI, (**G**): axial CE pancreatic parenchymal phase FST1W1, (**H**): axial CE portal venous phase FST1W1. Primary pancreatic lymphoma (focal form) in a 26-year-old patient with obstructive jaundice. CT revealed a hypovascular mass in the head of the pancreas (thin arrows in (**A**,**B**)), with minimal bile (arrowhead in (**A**)) and MPD dilatation (thick arrow in (**A**)), no distal parenchymal atrophy and abutment of the superior mesenteric vein (arrowhead in (**B**)). Note in the T2WI a homogeneous slightly hyperintense mass (arrow in (**C**)) with marked diffusion restriction (arrows in (**D**,**E**)) and its hypovascularity following intravenous contrast administration. Considering the tumour size, its homogeneity, marked diffusion restriction, growth pattern and hypovascularity with minimal MPD and biliary dilatation, lymphoma was one of the top possibilities on the differential diagnosis list. Note a small haematoma in the center of the mass (arrows on (**F**–**H**)) secondary to a EUS-guided biopsy, which concluded Burkitt lymphoma. The patient was successfully treated with chemotherapy, obtaining a complete remission.

**Figure 17:**
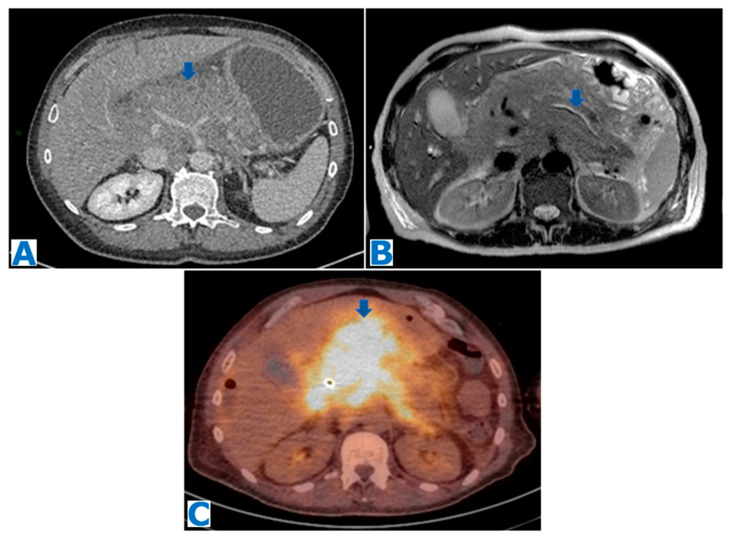
(**A**): axial CE portal venous phase CT, (**B**): axial T2WI, (**C**): FDG-PET-CT. Primary pancreatic lymphoma (diffuse form) in a 65-year-old alcoholic patient referred to our institution after being diagnosed with a pancreatic mass. An ill-defined infiltrating pancreatic mass is observed (arrow in (**A**)), with no biliary or pancreatic duct dilatation (observe the MPD’s normal appearance, arrow in (**B**)). The mass shows an intense hypermetabolic uptake on the FDG-PET-CT (arrow in (**C**)). Biopsy revealed a high-grade PPL with diffuse big cell B lymphoma and Burkitt-like components.

**Figure 18:**
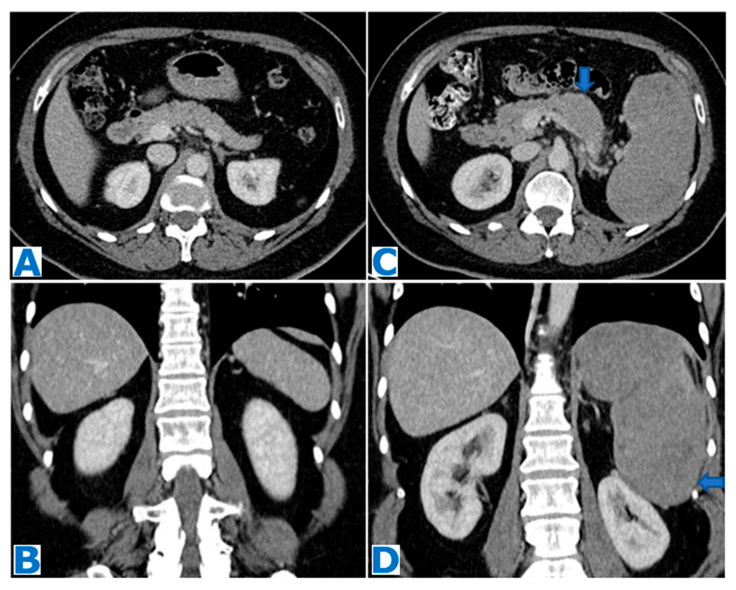
Images from 2017, (**A**,**B**): axial and coronal CE portal venous phase CT MPR. Images from 2022, (**C**,**D**): axial and coronal CE portal venous phase CT MPR. Secondary pancreatic lymphoma in a 60-year-old patient with a known glomus jugulotympanicum paraganglioma on surveillance. Note the normal appearance of pancreas and the spleen (**A**,**B**) on the prior CT. Abdominal adenopathies were found (not shown). Observe the spleen enlargement (arrow in (**D**)), with focal lesions. Hepatoduodenal and retroperitoneal adenopathies were also found (not shown). Note the isoenhancing mass within the body of the pancreas (arrow in (**C**)). EUS-guided FNB revealed a diffuse large B-cell SPL.

### 4.7. Pancreatic Metastases (Figure 19, Figure 20, Figure 21 and Figure 22)

Metastases to the pancreas are uncommon, only accounting for 2–5% of pancreatic malignancies [273]. They mostly occur secondary to intra-abdominal tumours [274], including RCC, colon and gastric cancer [275,276], although lung cancer also ranks high among the most frequent sites of origin [276].

PM may invade the epithelium of the pancreatic duct and mimic PDAC symptoms, namely, jaundice and abdominal pain as the most common presenting signs [276,277]. However, PM may also be asymptomatic and incidentally identified during the initial workup of the primary tumour or during surveillance. There may be a latency period from the diagnosis of the primary tumour to the detection of PM, which in the case of clear cell renal or breast carcinoma may be quite long, up to 21 years after surgery of RCC [276,277,278].

PMs are commonly associated with widespread disease, at a late stage, and more than 90% of patients have extrapancreatic disease [279]. However, it should be noted that in more than half of PM cases, the pancreas is the only organ metastatically involved [280], especially in RCC [281].

There is no location predilection within the pancreas [282]. In the particular case of PM from lung cancers, the head seems to be a favoured site as 76% of small cell lung carcinomas, the histological type mostly associated with PM [283], arise there [284].

Cancer antigens have little diagnostic reliability [285]. In an analysis of series with 192 cases in total, CA 19-9 was elevated in 8–28% of cases, but this may be related to the gastrointestinal origin of most of the primary tumours included and unrelated to PM [286].

Three patterns of metastatic involvement have been described. The most common appearance (50–75%) is the single pattern, depicting a solitary, localised and well-defined lesion. The second most common is the diffuse infiltration that causes a generalised enlargement of the pancreas (15–44%). The remaining pattern (5–10%) is represented by several nodules, which can coalesce into larger masses [280,287].

Dilatation of the main pancreatic or bile ducts is uncommon [288].

PMs typically appear hypointense in FST1WIs compared to normal parenchyma and may show moderate hyperintensity in T2WIs [289] although they may also appear hypointense, especially in the diffuse infiltration pattern. The behaviour after intravenous contrast injection relates to size: even though most of the lesions are hypovascular, lesions smaller than 1.5 cm may be hypervascular and larger lesions may show a rim of enhancement due to central necrosis [290,291]. This peripheral enhancement pattern has been described as a frequent finding (41%) [263,292], especially in PMs from RCC. The rationale behind this enhancement pattern is that the periphery of the lesion receives more blood than the center, since PMs nurture themselves by parasitising blood supply from the surrounding parenchyma.

As in any other organ, PM features resemble those of the primary tumour, e.g., PMs from RCC are often hypervascular [293]. PMs from melanoma, due to the paramagnetic effect of melanin, show a high signal intensity in T1WIs and low signal intensity in T2WIs [294]. PMs from dermatofibrosarcoma are usually hypointense in T1WIs, slightly hyperintense in T2WIs and show a spoke wheel-like enhancement [295].

At least one third of PMs are misdiagnosed as primary tumours [296]. Differential diagnosis of hypervascular PM should include primary pancreatic NET, intrapancreatic accessory spleen and vascular lesions [292]. Hypovascular PMs need to be differentiated from PDAC, lymphoma and focal pancreatitis [297,298]. Peripheral enhancement is a useful sign to differentiate PM from PDAC [263]; other distinguishing features are absence of dilatation of the upstream pancreatic duct and/or bile ducts, parenchymal atrophy and absence of vessel involvement [293,294].

The treatment of choice in eligible patients is pancreatic metastasectomy. However, its success depends on the biology of the primary tumour. According to most large studies, the best long-term survival predictor is the type of cancer [275,299]. PMs from RCC achieve the best outcome (61% 5-year survival) [274] and surgery is the treatment of choice if all metastatic lesions can be resected, although there is a high rate of recurrence (33–42% of patients who undergo pancreatic metastasectomy). On the other end of the spectrum, lung carcinoma is associated with the worst survival (0%) [300].

**Figure 19:**
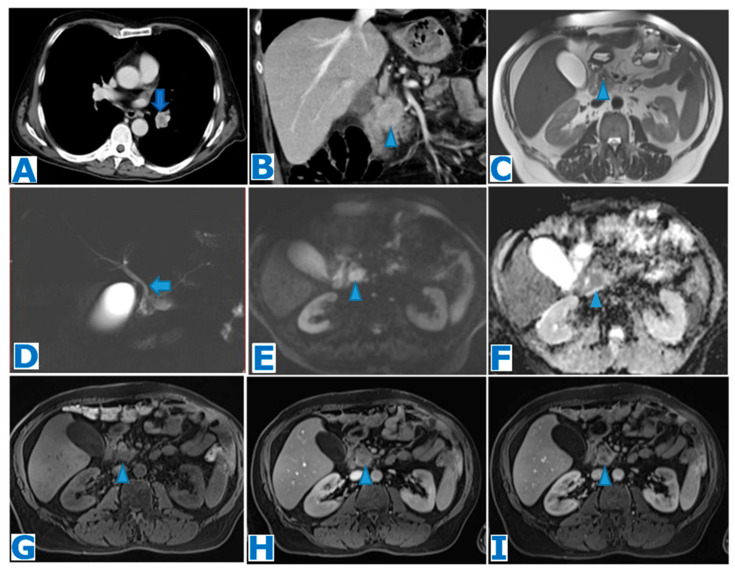
(**A**): axial CE-CT, (**B**): coronal MPR CE portal venous phase CT, (**C**): axial T2WI, (**D**): MRCP, (**E**): DWI, (**F**): ADC, (**G**): axial NCE FST1WI, (**H**): axial CE arterial phase FST1WI, (**I**): axial CE portal venous phase FST1WI. Solitary PM from lung adenocarcinoma in a 56-year-old patient with advanced stage disease and hyperbilirubinemia. Observe the primary tumour within the left hilum (arrow in (**A**)). The patient also presented peritoneal and bone metastases, not shown. A mass was found within the head of the pancreas (arrowhead in (**B**)), with no MPD dilatation (arrowhead in (**C**)). However, the lesion was associated with discreet common bile duct dilatation (arrow in (**D**)). Observe the diffusion restriction (arrowhead in (**E**,**F**)) and the progressive peripheral enhancement following intravenous contrast administration (arrowhead in (**G**–**I**)) with central necrosis. Note the resemblance to the primary tumour (arrow in (**A**)). EUS-guided FNB confirmed a PM from an adenocarcinoma of pulmonary origin.

**Figure 20:**
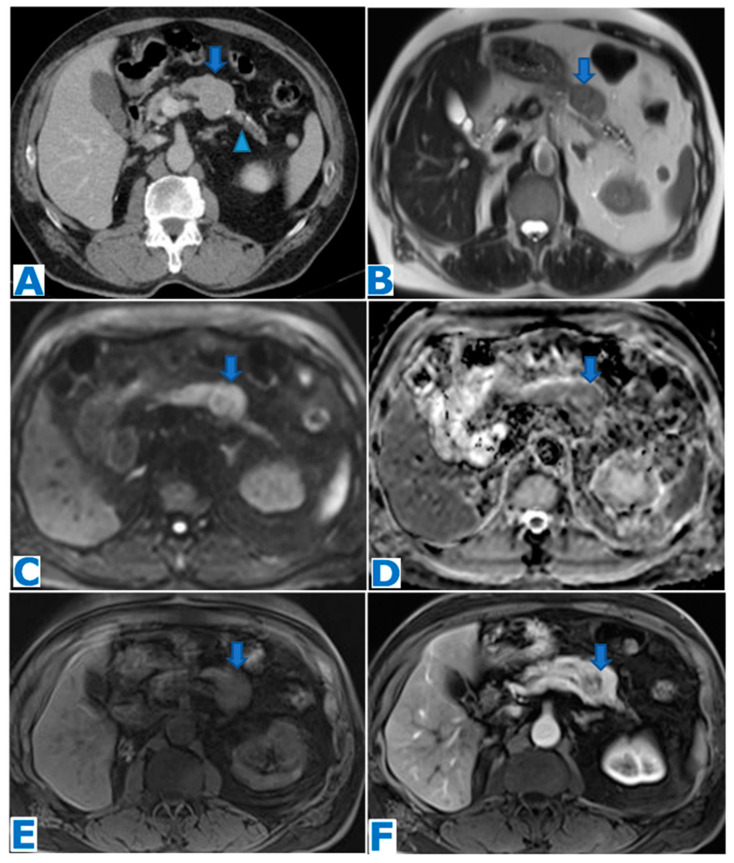
(**A**): axial CE-CT, (**B**): axial T2WI, (**C**): DWI, (**D**): ADC, (**E**): axial NCE FST1W1, (**F**): axial CE arterial phase FST1W1. Solitary PM from a known RCC discovered during follow-up in a 56-year-old patient who underwent a right nephrectomy 10 years prior. A homogeneous mass was found within the body of the pancreas (arrow in (**A**,**B**)), with discrete pancreatic duct dilatation and distal parenchymal atrophy (arrowhead in (**A**)). The mass showed diffusion restriction (arrow in (**C**,**D**)) and marked peripheral enhancement (arrow in (**E**,**F**)). An EUS-guided FNB showed rare epithelial cells and concluded haemorrhagic cyst. Given the discordance between the images and the histological report, the MDT decided to perform a left pancreatectomy and the histological examination concluded RCC metastasis.

**Figure 21:**
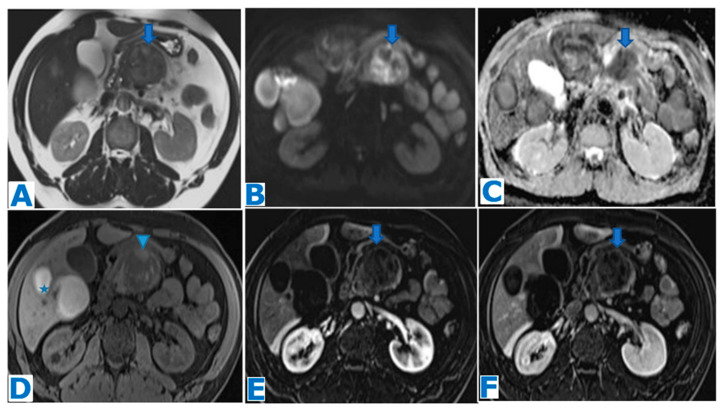
(**A**): axial T2WI, (**B**): DWI, (**C**): ADC, (**D**): NCE FST1W1, (**E**): CE arterial phase FST1W1 subtraction, (**F**): CE portal venous phase FST1W1 subtraction. PM from a known malignant skin melanoma in a 54-year-old patient who presented with acute abdominal pain. A heterogeneous mass was found within the body of the pancreas (arrow in (**A**)), showing diffusion restriction (arrow in (**B**,**C**)) and no pancreatic duct dilatation. The lesion showed hyperintense content in T1WIs (arrowhead in (**D**)) compatible with melanin, with scarce enhancement after intravenous contrast administration (arrow in (**E**,**F**)). There were also several melanin-containing hepatic lesions (* in (**D**)). Diagnosis of pancreatic and hepatic metastases was proven by biopsy.

**Figure 22:**
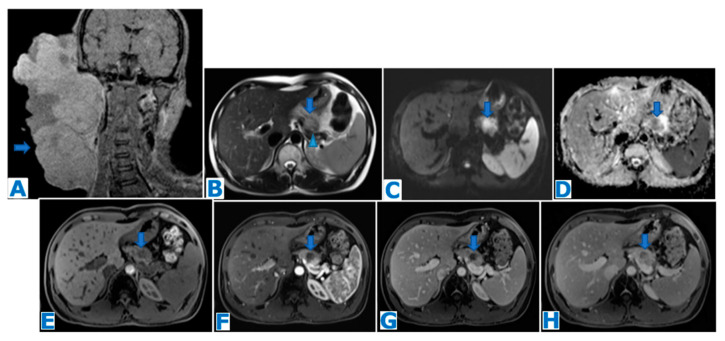
(**A**): image from 2016: coronal CE T1WI. Images from 2021: (**B**): axial T2WI, (**C**): DWI, (**D**): ADC, (**E**): NCE FST1WI, (**F**–**H**): axial CE dynamic FST1WI (pancreatic parenchymal, portal venous and delayed venous phase). PM from a facial DFSP in a 35-year-old patient who presented with elevated pancreatic enzymes on a check-up, five years after the primary tumour diagnosis (arrow in (**A**)). Five solid lesions were found within the pancreas, of which only one is shown, slightly hyperintense in T2WIs (arrow in (**B**)) with minimal MPD dilatation (arrowhead in (**B**)), diffusion restriction (arrow in (**C**,**D**)) and a hypovascular behaviour following intravenous contrast administration with progressive enhancement (corresponding to the fibrous content) (arrow in (**E**–**H**)). Histological examination following EUS-FNB concluded PM from DFSP.

## 5. Conclusions

Several rare focal and diffuse lesions may be found in the pancreas, either incidentally discovered, related to specific or, most frequently, non-specific clinical symptoms and biological abnormalities, or in the setting of a known oncologic condition. These lesions are associated with different behaviours, which range from benign to very aggressively malignant and, therefore, they are associated with different prognosis. Cross-sectional imaging findings combined with the clinico-biological setting contribute substantially to achieving the correct diagnosis. Typical imaging features related to the appearance of the lesion on cross-section imaging modalities in addition to indirect associated signs, such as the presence/absence of biliary and/or pancreatic duct dilatation, invasion of adjacent organs, peripancreatic vascular involvement or loco-regional lymph nodal invasion together with the presence of distant metastases, are crucial to correctly address the diagnosis.

Nevertheless, challenging cases occur, in which imaging features remain indeterminate and there is no typical clinical or biological presentation, and thus EUS-FNA/FNB is required to obtain histologically proven confirmation of the nature of the lesion, which is mandatory for optimal patient management.

## Figures and Tables

**Table 1:** Classification of rare solid pancreatic lesions.

Benign	Potentially Malignant	Malignant
Intrapancreatic splenic tissue	Solid pseudopapillary tumour	Acinar cell carcinoma
Tuberculosis	Schwannoma	Undifferentiated carcinoma with osteoclastic-like giant cells.
Solid serous cystadenoma	Purely intraductal neuroendocrine tumour	Adenosquamous carcinoma
	Fibrous solitary tumour	Colloid carcinoma
		Primary leiomyosarcoma
		Lymphoma (primary and secondary)
		Metastases

**Table 2:** No. of cases/incidence/prevalence of rare solid pancreatic lesions depicted.

Rare Solid Pancreatic Lesions	No. of Cases/Incidence/Prevalence
Intrapancreatic splenic tissue	61 cases/3000 autopsies
Tuberculosis	116 cases
Solid serous cystadenoma	22 cases
Solid pseudopapillary tumour	2% of all exocrine pancreatic neoplasms
Schwannoma	<80 cases reported
Purely intraductal neuroendocrine tumour	7 cases reported
Fibrous solitary tumour	29 cases reported
Acinar cell carcinoma	<2% of all primary pancreatic neoplasms
Undifferentiated carcinoma with osteoclasic-like giant cells	<1% of all malignant pancreatic neoplasms
Adenosquamous carcinoma	0.38–10% prevalence
Colloid carcinoma	1% of all pancreatic tumours
Primary leiomyosarcoma	0.1% of malignant pancreatic neoplasms
Primary lymphoma	<0.5% of all primary pancreatic neoplasms, 1% of all extranodal lymphomas
Secondary lymphoma	30% cases of extranodal lymphoma
Metastases	2–5% of pancreatic malignancies

## Supplementary Materials

The following supporting information can be downloaded at: https://www.mdpi.com/article/10.3390/diagnostics13162719/s1, Table S1: Benign Lesions; Table S2: Potentially Malignant Lesions; Table S3: Malignant Lesions.
