# Supplementary material for: Rare Solid Pancreatic Lesions on Cross-Sectional Imaging
Source: Diagnostics (Basel). 2023 Aug 21;13(16):2719. doi: 10.3390/diagnostics13162719 (PMC10453474; doi:10.3390/diagnostics13162719)
Supplement: Supplementary file 1 [file diagnostics-13-02719-s001.zip › diagnostics-2560475-supplementary.pdf]

Table S1. Summary of rare benign solid pancreatic lesions.

	IST	PT	SSCA
**Demographics**	?	4th- 5th decade.<br>Immunosuppression.	Women 7th decade.
**Laboratory**	-	-	-
**Prognosis**	Benign	Good	Benign
**Location**	Tail	Body, head	?
**Appearance**	Well-defined.	Well-defined.<br>Focal mass.<br>Multiple small nodules.<br>Diffuse involvement.	Well-defined
**Enhancement**	Hypervascular.<br>Same as spleen.<br>Zebra pattern.	Peripheral enhancement.	Rapid arterial enhancement.<br>Isodensity/intensity portal phase.
**Calcification**	-	++	-
**Haemorrhagic content**	-	-	++
**Cysts**	-	++	-
**Necrosis**	-	-	-
**MPD dilatation**	-	Rare	Rare
**Vascular invasion**	-	-/+	-
**TIP**	Prior splenic surgery or trauma.<br>High DWI SI.	Peripancreatic necrotic adenopathies.	MRCP: cysts
**Differential diagnosis**	pNEN<br>SPT<br>Hypervascular metastasis.	***Solid appearance:***<br>• PDAC<br>• Lymphoma<br>• Metastasis<br>***Cystic appearance:***<br>• Cystadenoma<br>• Pseudocyst<br>• Abscess<br>***Diffuse involvement:***<br>• Acute pancreatitis.	pNEN<br>SPT<br>Hypervascular metastasis.

IST Intrapancreatic splenic tissue, *PDAC* Pancreatic ductal adenocarcinoma, *pNEN* Pancreatic neuroendocrine tumour, *PT* Pancreatic tuberculosis, *SPT* Solid pseudopapillary tumour, *SSCA* Solid serous cystadenoma

Table S2. Summary of rare potentially malignant solid pancreatic lesions.

	SPT	PS	INEN	PSFT
**Demographics**	Women < 40 y.o.<br>Men: older, aggressive behaviour.	5th decade.<br>10% association NF2.<br>Rarely NF1 and risk of malignancy.	?	6th decade
**Laboratory**	-	-	Non-functioning pNEN.	Recurrent hypoglucemia.
**Prognosis**	Good if R0 resection,<br>even if metastatic.<br><br>Malignant behaviour 10-15%.	Good.<br><br><br>Malignant transformation possible.	?	Good if R0 resection.<br><br>12-22% malignant.
**Location**	Tail	Head	?	Head
**Appearance**	Well-defined.<br>Large.<br>Heterogeneous.<br><br>Homogeneous if small	Well-defined.<br>Solid-cystic.	Purely intraductal.<br><br>Distal pancreas atrophic.	Well-defined.<br>Large.<br>Heterogeneous.<br><br>Homogeneous if small
**Enhancement**	++	Determined by proportion solid/cystic components	+++	++
**Calcification**	+	-/+ if large	?	++if large
**Hemorraghic content**	++	-/+ if large	?	+if large
**Cysts**	++	-/+	?	++if large
**Necrosis**	++	-/+ if large	?	+++ if large
**MPD dilatation**	-/+ if malignant	-	++	-/+ if malignant
**Vascular invasion**	+if malignant	+if malignant	-	-
**TIP**	Fibrous pseudocapsule.<br>Fluid-fluid levels.	Hypermetabolic on PET-CT even if benign.	May be obscured if causing acute pancreatitis.	Hypervascular.<br>Adenopathies rare.<br>PET-CT not useful for malignancy.
**Differential diagnosis**	pNEN.<br>Hypervascular metastasis.	SPT<br>pNEN<br>SSCA	Chronic pancreatitis.	pNEN<br>PPLM<br>GIST<br>PEComa<br>SPT

GIST gastrointestinal stromal tumour, *INEN* Intraductal Neuroendocrine Neoplasm, *PEComa* perivascular epithelioid cell tumour, *pNEN* Pancreatic neuroendocrine tumour, PPLM Primary pancreatic leiomyosarcoma, *PS* Pancreatic schwannoma, *PSFT* Pancreatic solitary fibrous tumour, *SPT* Solid pseudopapillary tumour, *SSCA* Solid serous cystadenoma.

Table S3. Summary of rare malignant solid pancreatic lesions.

	ACC	UCOGC	PASC	CC	PPLM	PPL	SPL	PM
**Demographics**	Men>women.<br>Bimodal:<br>8-15 y.o<br>7th decade	Women>>men<br>Middle aged and elderly.	Men>women.<br>6th decade.	7th decade.	5th decade.<br>East Asian ethnicity.	Men > women.<br>5th decade.<br>Immunosuppression.	Known lymphoma.<br>More frequent than PPL.	Known primary tumour: RCC, gastric, breast, lung.
**Laboratory**	↑ Lipase.<br>↑Alpha-fetoprotein.<br>Peripheral eosinophilia.	N/↑CEA<br>N/↑CA19-9	↑CEA<br>↑CA19-9<br>Hypercalcemia	↑CEA<br>↑CA19-9	-	↑LDH	↑LDH	-
**Prognosis**	5-year survival rate 50%	5-year survival rate > 50%.<br>Variable.	Median overall survival after surgery 1 year.	5-year survival rate 57%.	48 months	Long-term regression/remission.	Long-term regression/remission.	Depends on primary tumour.
**Location**	-	Body-tail	Head	Head	-	Head	-	-
**Appearance**	Large.<br>Well-defined.<br>Exophytic.	Large.<br>Locally aggressive.	Round.<br>Lobulated.	Large.<br>Lobulated.<br>Ill-defined.<br>Intraluminal if IPMN-related.	Large.<br>Heterogeneous.	*Focal pattern:*<br>Homogeneous<br>Large<br><br>*Other:*<br>Diffuse<br>Peripheral<br>Multinodular	Peripancreatic adenopathy with pancreas invasion.<br><br>Same patterns as primary pancreatic lymphoma.	Single.<br>Multiple.<br>Diffuse.
**Enhancement**	-Arterial phase<br>+ Portal phase	-/+	+	Progressive and delayed.<br>Sponge-like.	+<br>If large:<br>+peripheric<br>- central	Limited.<br>Progressive.<br>Delayed.	+	Like primary tumour
**Calcification**	-/+ if large	-/+	-	++	-	-	-	Like primary tumour
**Hemorraghic content**	-/+ if large	-/+	-	-	-/+ (if large)	-	-	Like primary tumour
**Cysts**	-/+(if large)	+	-	-	-/+(if large)	-	-	-
**Necrosis**	-/+ if large	-/+	++	-	-/++ if large	-	-	Like primary tumour
**MPD dilatation**	-	+	+	-/+	-	-	-	-
**Vascular invasion**	-/+	+	-/+	-/+	-/+	-	-	-
**TIP**	May be exophytic.<br>Enhancing capsule.<br><br>Adenopathies and hepatic	Invasive.<br><br>Adenopathies and metastases rare.	Central high SI T2WI.<br>Enhancing capsule.<br>Locally advanced / metastatic.	↑ High T2 SI.<br><br>Salt&pepper pattern.	Adenopathies rare.<br><br>Metastases frequent at presentation.	High DWI signal.<br><br>If infrarenal retroperitoneal adenopathy,	Known or suspected lymphoma.	Advanced disease.<br><br>Long latency.

	metastases frequent at presentation.					PDAC ruled out.		
**Differential diagnosis**	PDAC.<br>pNEN.<br>SPT.<br>Pancreatoblastoma.	PDAC.<br><br>Mucinous carcinoma.<br><br>SPT.<br>pNEN.<br>Pseudocyst.	PDAC	IPMN<br><br>Mucinous carcinoma.	PDAC<br>pNEN	PDAC<br>***Diffuse pattern:***<br>Acute pancreatitis.<br>***Peripheral pattern:***<br>AIP.<br>***Multinodular pattern:***<br>PM.<br>Multifocal AIP.	PDAC<br>PPL<br>Same as PPL	***Hypervascular***<br>pNEN<br>IPST<br>Vascular<br><br>***Hypovascular***<br>PDAC<br>PPL<br>AIP

ACC Acinar cell carcinoma, *AIP* Autoimmune pancreatitis, *CC* Colloid carcinoma, *IPMN* Intraductal papillary mucinous neoplasm, *PASC* Pancreatic adenosquamous carcinoma, *PDAC* Pancreatic ductal adenocarcinoma, *pNEN* Pancreatic neuroendocrine tumour, *PM* Pancreatic metastases, *PPL* Primary pancreatic lymphoma, *PPLM* Primary pancreatic leiomyosarcoma, *SPL* Secondary pancreatic lymphoma, *SPT* Solid pseudopapillary tumour, UCOGC Undifferentiated carcinoma with osteoclastic-like giant cells.
